# Smart Biointerfaces via Click Chemistry-Enabled Nanopatterning of Multiple Bioligands and DNA Force Sensors

**DOI:** 10.1021/acsami.4c00831

**Published:** 2024-04-18

**Authors:** Ali Shahrokhtash, Duncan S. Sutherland

**Affiliations:** † Interdisciplinary Nanoscience Center, Aarhus University, Gustav Wieds Vej 14, 8000Aarhus C, Denmark; ‡ The Centre for Cellular Signal Patterns (CellPAT), Gustav Wieds Vej 14, 8000 Aarhus C ,Denmark

**Keywords:** protein nanopatterning, surface click chemistry, 3T3 cells, hole-mask colloidal lithography, DNA force sensors

## Abstract

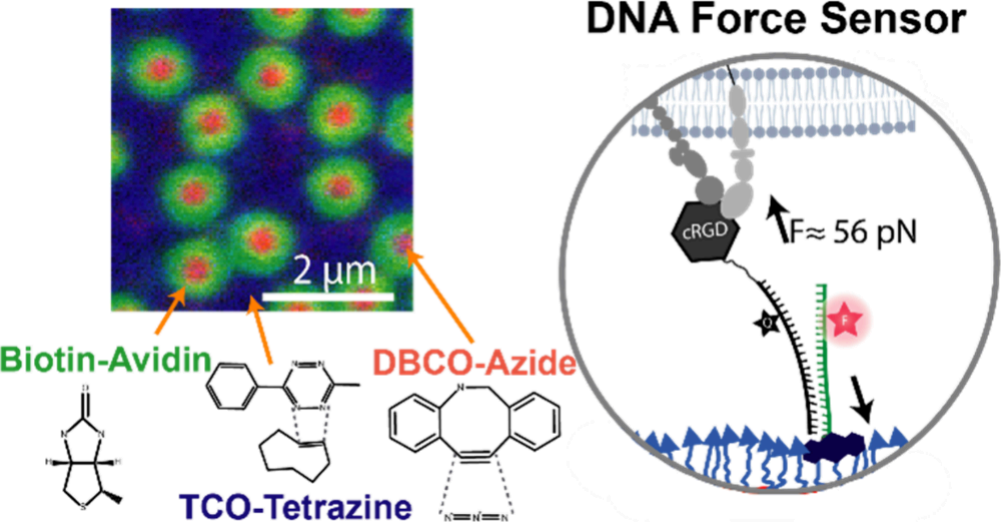

Nanoscale biomolecular placement is crucial for advancing cellular signaling, sensor technology, and molecular interaction studies. Despite this, current methods fall short in enabling large-area nanopatterning of multiple biomolecules while minimizing nonspecific interactions. Using bioorthogonal tags at a submicron scale, we introduce a novel hole-mask colloidal lithography method for arranging up to three distinct proteins, DNA, or peptides on large, fully passivated surfaces. The surfaces are compatible with single-molecule fluorescence microscopy and microplate formats, facilitating versatile applications in cellular and single-molecule assays. We utilize fully passivated and transparent substrates devoid of metals and nanotopographical features to ensure accurate patterning and minimize nonspecific interactions. Surface patterning is achieved using bioorthogonal TCO-tetrazine (inverse electron-demand Diels–Alder, IEDDA) ligation, DBCO-azide (strain-promoted azide–alkyne cycloaddition, SPAAC) click chemistry, and biotin–avidin interactions. These are arranged on surfaces passivated with dense poly(ethylene glycol) PEG brushes crafted through the selective and stepwise removal of sacrificial metallic and polymeric layers, enabling the directed attachment of biospecific tags with nanometric precision. In a proof-of-concept experiment, DNA tension gauge tether (TGT) force sensors, conjugated to cRGD (arginylglycylaspartic acid) in nanoclusters, measured fibroblast integrin tension. This novel application enables the quantification of forces in the piconewton range, which is restricted within the nanopatterned clusters. A second demonstration of the platform to study integrin and epidermal growth factor (EGF) proximal signaling reveals clear mechanotransduction and changes in the cellular morphology. The findings illustrate the platform’s potential as a powerful tool for probing complex biochemical pathways involving several molecules arranged with nanometer precision and cellular interactions at the nanoscale.

## Introduction

The precise arrangement of multiple biomolecules on surfaces is essential for biosensing, tissue engineering, and cellular research applications.^1−3^ In particular, the organization of ligands at the subcellular and molecular scale enables studies of cellular adhesion and signaling processes.^4−6^ A range of patterning techniques have been developed to arrange these molecules, and micropatterning techniques have had increased accessibility recently, allowing the formation of patterns over large areas within the dimensions of the cell to form constrained adhesion regions using methods such as microcontact printing.^7^ Similarly, the generation of multicomponent biomolecule micropatterns by maskless photolithography-based approaches using digital micromirror devices and commercialization of those has further enhanced the availability of biological micropatterning.^8^ However, the utility of these approaches at the subcellular scale still remains limited.

Nanolithography methods such as electron-beam lithography have been employed for the direct writing of multiple proteins to control the organization of biomolecules at the subcellular and molecular scale. These approaches rely on incorporating the proteins in polymers to tolerate the non-native patterning conditions (e.g., vacuum)^9^ with the freedom to generate arbitrary patterns. Similarly, chemical contrast patterns for indirect writing methods to immobilize biomolecules in the patterned regions with 10 nm precision have been developed using this technology.^5^ However, these serial pattern generation processes result in low throughput and high-fabrication costs.

Other serial nanopatterning approaches, such as dip-pen nanolithography,^10^ are commonly used to generate biomolecular patterns, both by direct and indirect writing of the biomolecules. Remarkably, parallelization of the cantilever pens from 1 to 55,000,^11^ as well as combining soft-lithography to fabricate large stamps of elastomeric pyramid shape in polymer-pen lithography, has increased the throughput of this approach to pattern mm^2^ regions with repetitive patterns for low-throughput experiments.^12^

Recent advances in DNA nanotechnology and the emergence of DNA origami in cell culture studies have allowed the patterning of multiple biomolecules with sub-10 nm precision. Nano- and micropatterns of immobilized DNA origami on surfaces are a promising approach for presenting multiple ligands with nanometer precision.^4,13,14^

However, the limited size of the origamis (typically up to 100 × 100 nm) constrains the number and dimensions of presented ligands. Furthermore, DNA origami’s very high negative charge can lead to nonspecific binding, interfere with cellular interactions, and require a nonphysiological concentration of ions to maintain their structural integrity. Likewise, the reduced stability in complex physiological media can limit the duration of in vitro experiments on these biointerfaces to a few hours, emphasizing the need to introduce protecting modifications such as passivation and charge neutralization to the origamis. However, these modifications can interfere with the presentation of the patterned ligands.^15,16^

As an alternative to the above, bottom-up fabrication methods such as block copolymer micelle nanolithography^17,18^ and colloidal lithography have emerged as high-throughput options for nanoscale patterning without requiring specialized instruments.^19−21^

To form biomolecular patterns with high fidelity and stability, preventing the nonspecific binding of proteins and biomolecules driven by intramolecular forces and entropic effects is crucial.^22^ These nonspecific interactions lead to the attachment of unwanted and often unidentified biomolecules at the surfaces, which can interfere with studying cellular signaling processes using patterned substrates.

When densely assembled on surfaces, a specific class of inert, hydrophilic polymers can diminish surface energy and restrict nonspecific protein adsorption. Poly(ethylene glycol) (PEG) and poly(2-methyl-2-oxazoline) (PMOXA), organized in a brush-like configuration, serve as archetypal examples.^23,24^ Such polymers are widely used in the surface functionalization of materials for drug delivery and preventing the nonspecific binding of proteins and cells on surfaces.^25,26^

These polymers can be readily affixed to surfaces through grafting-to, grafting-from techniques and via commercially available random block copolymers such as PLL-*g*-PEG or PAcrAm-*g*-PEG.^27−31^ While high polymer density can be achieved through any of these strategies, the latter facilitates rapid and easy polymer deposition from aqueous solutions. These random block copolymers offer the flexibility to tailor the grafting ratio and the presence of functional adhesive groups, thereby fine-tuning surface adhesion. Serrano et al. demonstrated that these polymers could be chemically stabilized over long term by functionalizing them with covalent siloxane and strong coordinating nitrodopamine (ND) moieties.^30^

Beyond their antifouling properties, these random block copolymers can be engineered with orthogonal biospecific tags, permitting selective biomolecular adhesion via complementary tagging. Notable successful examples of such tags are biotin–avidin and strain-promoted azide–alkyne cycloaddition (SPAAC) covalent click chemistry.^31−34^ Careful choice of biospecific tags plays a vital role in preventing the nonspecific interaction of biomolecules with the passivated surfaces. Antifouling brushes with biospecific tags against common motifs, such as specific amino acids, for instance, NTA-his tags may not consistently maintain a protein antifouling efficacy above 90%.^35^

Despite the range of techniques developed and the development of antifouling polymer coatings, nanometer patterning of multiple ligands while maintaining functional and stable surfaces against nonspecific binding remains a challenge in biological settings.

In this work, we present a metal-free platform for the rapid fabrication of antifouling surfaces capable of orthogonal conjugation of three biomolecules within nanometer-scale patterns (below 1000 nm) using two orthogonal click chemistry approaches and biotin–avidin interactions.

The method allows for the conjugation of diverse molecules, such as DNA and proteins, that are suited for in vitro studies involving receptor-proximity effects and the formation of complex extracellular matrices. Importantly, the antifouling nature of the fabricated surfaces minimizes biomolecule displacement by cellular or media-derived proteins, thus maintaining the integrity of the patterned regions. The biomolecular patterning approach can be integrated into standard cell culture well plate formats and applied to provide subcellular patterned biomolecular adhesive and signaling molecules for mechanistic studies or as patterned signals to drive cellular phenotypes. As a proof-of-principle cellular demonstration, we showed the applicability of the patterning approach to study proximity effects between growth factors and cellular adhesions using nanopatterns of arginylglycylaspartic acid (RGD) conjugated to DNA-based force sensors and immobilized epidermal growth factor (EGF) allowing the readout of mechanical events in cellular adhesions.

## Results and Discussion

Nanometer-precise molecular patterning, at scales smaller than cellular dimensions, holds a critical significance for understanding cellular interactions. Thus, we fabricated size-tunable protein nanopatterns with control over biomolecular adhesion with nanometer precision.

In subsequent sections, we outline methodologies to spatially control the adhesion of these biospecific antifouling polymers with nanometer precision. Specifically, we focus on the ability to pattern up to three orthogonal tags within submicron size-tunable regions on large-area (cm^2^) surfaces that are fully transparent and free of topographic features, which have been shown to alter the cellular response strongly.^36,37^

### Protein Nanopatterning on Passivated PEG Surfaces via Sacrificial Hole-Mask Resist

**Figure 1:**
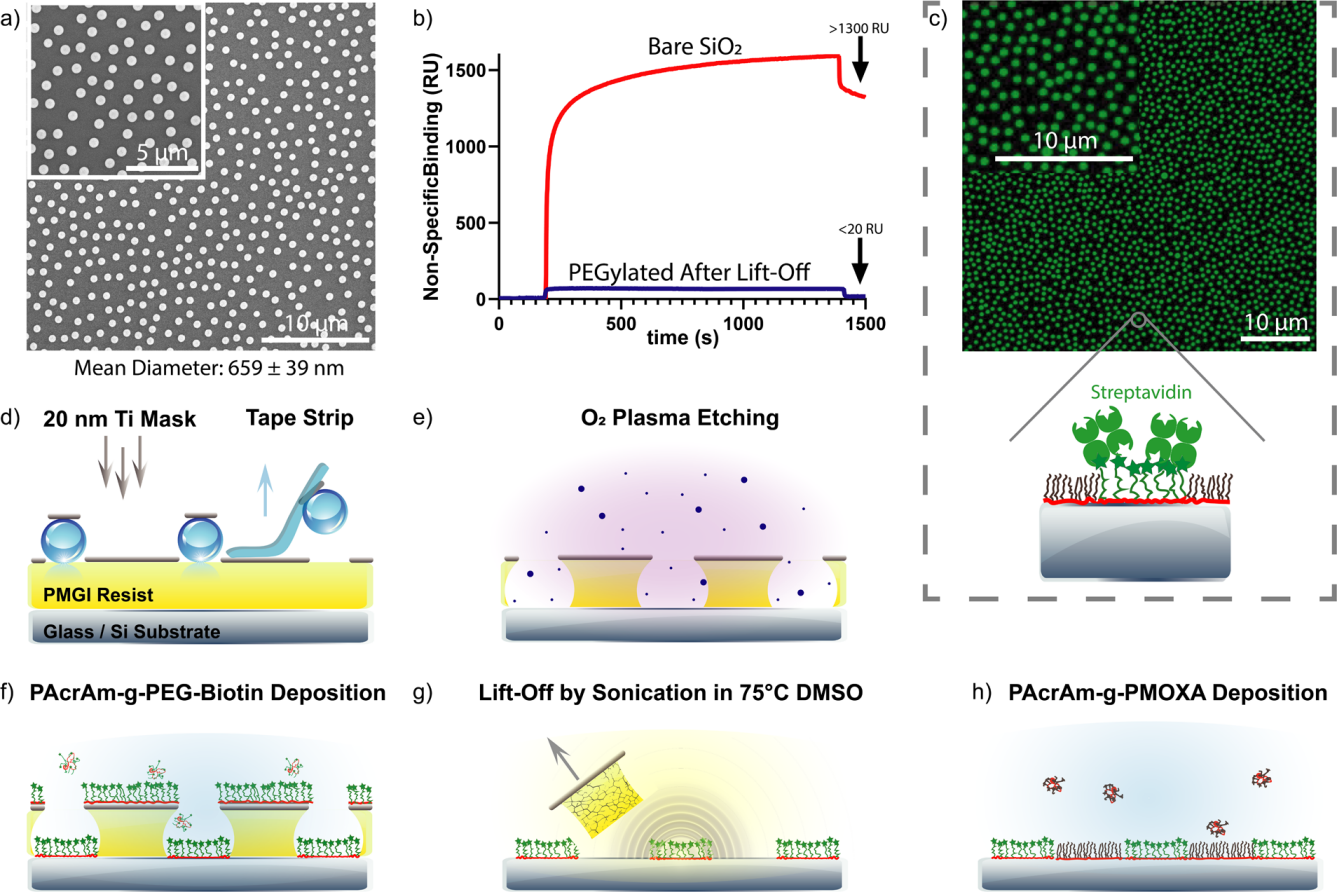
Fabrication of protein patterns. (a–e) Fabrication steps: (a) Deposition of hard Ti-mask on nanoparticles assembled on a PMGI resist layer and subsequent removal by tape stripping. (b) O_2_ plasma reactive ion etching removes the underlying resist layer, generating the hole mask. (c) Deposition of PAcrAm-*g*-PEG-Biotin, covering the substrate within the nanoapertures. (d) Complete removal of the sacrificial resist layer by sonication in 75 °C DMSO. (e) Deposition of antifouling PAcrAm-*g*-PMOXA, passivating the background region between the particles. (f) SEM images showing the characteristic sparse monolayer assembly of 600 nm (nominal size). The measured mean particle size is reported below the image. (g) SPR sensorgram showing nonspecific binding of BSA to the SiO_2_ vs PEGylated SiO_2_ layer after lift-off of the sacrificial PMGI resist layer. The arrow indicates the amount of nonspecifically bound BSA. (h) Fluorescence image showing 500 nm streptavidin nanopatterns and a schematic of streptavidin binding to the nanopatterned PEG-Biotin regions.

We employed colloidal nanoparticles as masks for nanoscale material deposition to expedite the fabrication of large-area (cm^2^) nanostructures. Specifically, we utilized hole-mask colloidal lithography (HCL), a scalable, straightforward, and cost-effective nanopatterning technique.^38^ This method applies an electrostatically assembled sparse colloidal nanoparticle monolayer to a spun-coated sacrificial resist layer (Figure 1a,f). Following metallic hard mask deposition and nanoparticle removal, the underlying resist layer is exposed to O_2_ reactive ion etching (RIE) to create holes for metal deposition directly onto the substrate (Figures S1 and S2). The fabricated structures’ surface density and dimensions are defined by the assembled nanoparticle monolayer and the projected shadow of the particles during metal deposition. The density of the structures can be tuned by changing the surface charge density of the nanoparticles, the ionic concentration during particle deposition, and the net surface charge of the surface.^39−41^

We then investigated the patterned deposition of antifouling PAcrAm-*g*-PEG-Biotin using the created hole masks. This polymer forms a dense PEG-Biotin brush while ensuring covalent surface adhesion through silane groups and electrostatic assembly via positively charged amine groups to the substrate’s negatively charged hydroxyl groups of the silicon dioxide (SiO_2_) layer. The resultant PEG-Biotin layer is expected to interact selectively only with the avidin protein family, including streptavidin (SA), ensuring specific binding to the biotin-tagged patterned region. To achieve a surface that exclusively binds avidin and avi-tagged proteins in the nanopatterned regions, replacing the spun-coated sacrificial resist layer with an antifouling polymer such as PAcrAm-*g*-PMOXA is necessary.

The fabrication workflow of this modified HCL protocol is outlined in Figure 1a–e. Initially, a sparse monolayer of nanoparticles is deposited, followed by a 20 nm titanium (Ti) mask, which is resistant to the O_2_ RIE. The particles shield the underlying areas from Ti deposition. Upon particle removal, the exposed regions remain uncoated and accessible. O_2_ RIE is then employed to selectively remove the sacrificial polydimethylglutarimide (PMGI) resist layer, creating holes down to the substrate (Figure 1b). These apertures serve as templating masks for PAcrAm-*g*-PEG-Biotin antifouling polymer deposition into the holes and onto the substrate (Figure 1c). Subsequent PMGI resist removal leaves behind nanoislands of PAcrAm-*g*-PEG-Biotin (Figure 1d). Finally, the pristine background region is coated with the antifouling PAcrAm-*g*-PMOXA polymer, restricting protein binding exclusively to Avi-tagged proteins within these nanopatterned regions, the dimensions of which correspond to the particles initially placed on the sacrificial resist layer (Figure 1e).

To successfully implement this strategy, several prerequisites must be met. First, the biospecific PAcrAm-*g*-PEG-Biotin polymer must be deposited from an aqueous solution without compromising the adhesion of the sacrificial resist layer, enabling it to act as an effective physical barrier during deposition. In the conventional HCL protocol, a 200 nm layer of poly(methyl methacrylate) (PMMA) serves as the sacrificial resist. While PMMA provides adequate adhesion during the aqueous phase sparse colloidal lithography step, its adhesion is weakened by the O_2_ RIE necessary to form the hole mask. As a result, incubation with antifouling polymers can dislodge the resist layer, leading to uncontrolled PAcrAm-*g*-PEG-Biotin polymer leakage beyond the patterned regions. To address this limitation without altering the surface chemistry of the thin glass coverslips used in our experiments, we substituted the PMMA resist with a PMGI-based resist of equivalent thickness. Notably, PMGI resists have been reported to offer stable adhesion yet permit clean lift-off.^42,43^ In our experimental conditions, the PMGI resist demonstrated superior substrate adhesion and was chosen for this nanofabrication strategy.

Subsequently, complete lift-off of the resist layer is required to enable robust adhesion of the antifouling PAcrAm-*g*-PMOXA polymer to form robust biomolecule patterns. This should happen under conditions that will preserve the integrity of the antifouling polymer and its biospecific tags. We discovered that 30 min ultrasonication in 75 °C dimethyl sulfoxide (DMSO) effectively eliminated the resist layer, as verified by X-ray photoelectron spectroscopy (XPS) (Figure S3). Optical and atomic force microscopy (AFM) further supported the complete removal of the resist, with the surface roughness (*R*_a_) matching that of O_2_ RIE-cleaned surfaces, approximately 0.1 nm.

For comparative purposes, PMMA lift-off in 40 °C acetone for an equivalent duration never yielded a *R*_a_ value below 0.4 nm, suggesting the substantial residual polymer presence on the substrate. Moreover, using surface plasmon resonance (SPR) chips that mimicked the nonpatterned background regions, we confirmed the functional integrity of the deposited antifouling PAcrAm-*g*-PMOXA polymer after PMGI resist removal (Figure 1g).

Upon refining this protocol, we validated the formation of fluorescently labeled SA nanopatterns via fluorescence microscopy (Figure 1h). Scanning electron microscopy (SEM) of biotinylated 40 nm gold nanoparticles selectively bound to the patterned SA regions provided additional confirmation (Figure S4).

Beyond dimensional attributes, the molecular density of the formed nanopatterns can be modulated by varying the number of adsorbed nanoparticles forming the mask or by adjusting the extent of biotin functionalization, potentially enabling the formation of single-molecule nanopatterns.^41^

The concept of patterning geometric regions with bioadhesive and nonadhesive regions through directed deposition of antifouling polymers using physical barriers has been previously illustrated by Falconnet et al., employing both supercellular-scale UV lithography and subcellular-scale nanoimprint lithography.^44,45^ Furthermore, HCL has been utilized to pattern individual SA molecules using a comparable methodology.^46^

However, fine-tuning the antifouling polymer deposition within the context of a modified HCL protocol represented a crucial step. This step serves as a foundation for subsequent fabrication stages, aimed at broadening the applicability of this strategy to pattern multiple bioorthogonal tags on a substrate that is entirely transparent and chemically passivated.

### Nanometer-Precise Dual-Biorthogonal Tag Proximity Positioning

To extend our capability for patterning multiple types of biomolecules with nanometer precision, we initially turned to selective molecular assembly patterning (SMAP). This technique leverages chemical contrast to direct material deposition into discrete regions.^47^ Utilizing the hole masks generated in the preceding step, we evaporated 2 nm-thick Ti or 20 nm-thick gold (Au) disks into these patterned features. Consequently, each hole mask contains two chemically distinct regions: an evaporated metallic disk corresponding to the original particle size and a ring-like undercut region of SiO_2_ adjacent to the disk. This bimodal chemical landscape within each hole mask permits spatially controlled deposition of biospecific PEG brushes.

Our initial efforts focused on the selective deposition of alkanethiols on Au and alkane phosphates on TiO_2_, aiming to prevent the adhesion of PLL-*g*-PEG molecules in these areas.^19,48,49^ However, when applied to PAcrAm-*g*-PEG polymers, which feature both electrostatic and covalent (siloxane-based) attachment to the surface, we found that the chemisorbed alkane layers were insufficient to prevent the strong adhesion of these polymers (data not shown). As a result, this strategy was deemed to be unsuitable for our requirements.

Subsequently, we examined catechols, such as ND, as a potential “grafting-to” candidate for PEG brush polymers, given their selective affinity for TiO_2_ over SiO_2_.^50^ Unfortunately, the coordination between ND and TiO_2_ was compromised during the lift-off process (data not shown). Additionally, PAcrAm-*g*-PEG molecules incorporating ND (in lieu of silane groups) and amine functionalities exhibited an overly strong adhesion to the SiO_2_ regions. Attempts to disrupt these possibly electrostatic interactions via high ionic concentrations or elevated pH conditions were unsuccessful; a considerable amount of polymer remained adhered to the surface (data not shown).

These setbacks indicate that attaining the requisite selectivity for dual biospecific tag deposition using chemical contrast alone remains challenging under the existing conditions.

**Figure 2:**
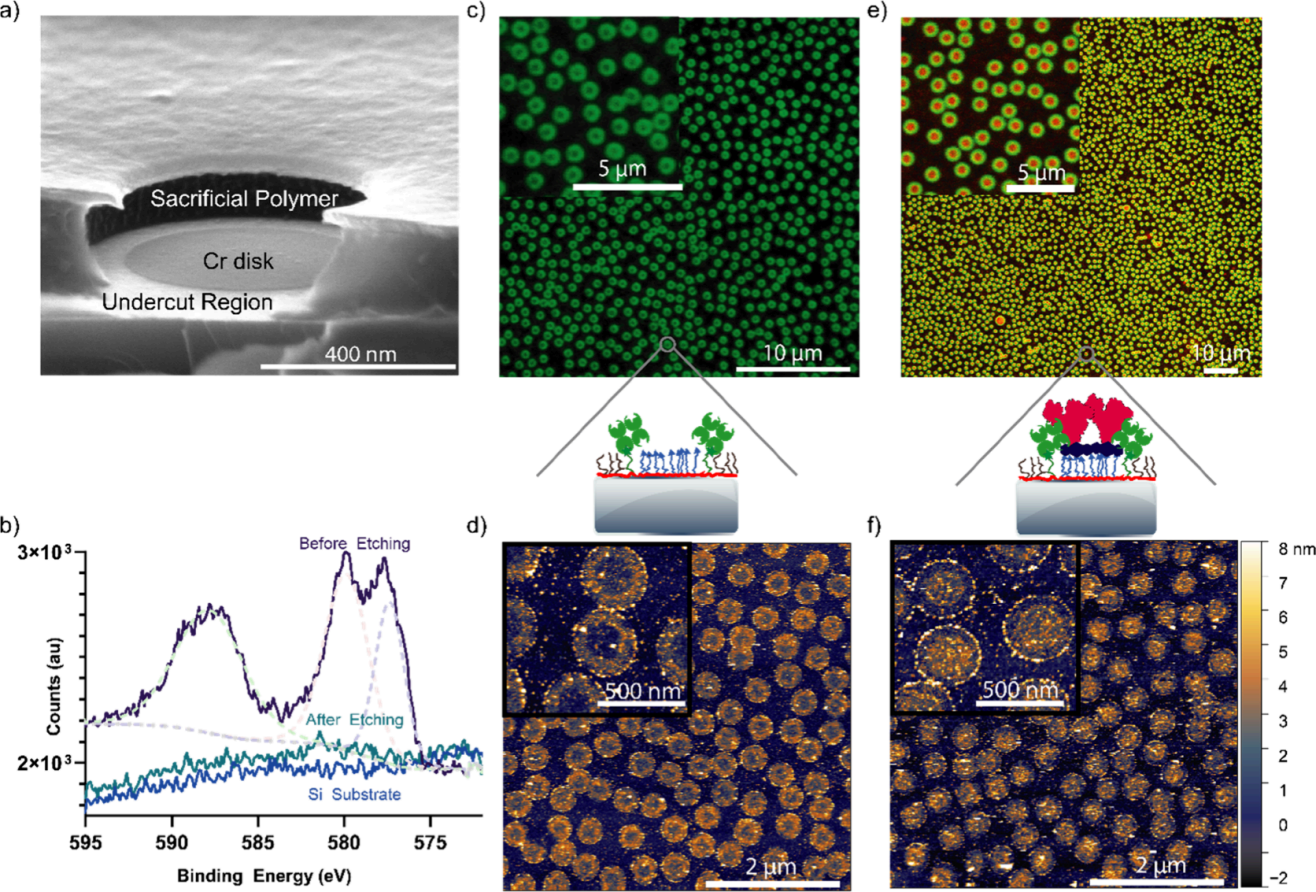
Orthogonal and site-specific patterning of the two ligands. (a) Side-view SEM of the hole mask, showing sacrificial Cr disks surrounded by the SiO_2_ undercut region and the sacrificial PMGI resist. (b) High-resolution Cr 2p 3/2 XPS spectra showing the removal of the sacrificial Cr disk after etching within the hole mask. (c) Fluorescence image showing 500 nm streptavidin-ring formation. (d) Liquid AFM scan of 200 nm nanopatterns after streptavidin incubation, showing a height increase in the ring region. (e) Fluorescence image of 800 nm nanopatterns incubated with both streptavidin (green) and DBCO-labeled BSA (green). (f) Similar to panel (d), incubated with both streptavidin and DBCO-labeled BSA, showing height increase in the “ring” and central “disk” regions, corresponding to protein binding in the defined regions.

Instead, to guide the targeted assembly of biospecific PEG brushes, we explored the introduction of a supplementary physical barrier, a 1 nm chromium (Cr) disk evaporated by physical vapor deposition within each hole mask (Figure 2a). This Cr disk was designed to remain in situ during the initial deposition of PEG layers to be subsequently eliminated via chemical etching. Such an approach hinged on two criteria: (1) the etchant must not compromise the structural integrity of the hole mask, constituted by the spun-coated PMGI resist layer, and (2) the previously deposited PAcrAm-*g*-PEG-Biotin layer must retain both its antifouling capabilities and its specific affinity for avidin.

To validate these premises, we first confirmed that the etchant did not alter the integrity of the hole mask or the PAcrAm-*g*-PEG-Biotin layer (Figure S5).^41^ Subsequently, we employed XPS to verify the complete removal of the Cr disks, a prerequisite for successfully depositing a secondary biospecific PEG brush. High-resolution XPS spectra targeting the Cr 2p 3/2 level confirmed the removal; the detectable peaks corresponding to the Cr disks disappeared postetching, reverting to baseline SiO_2_ levels (Figure 2b). This confirmed the full elimination of the Cr disk, thereby validating our etching process within the hole mask.

The remaining PAcrAm-*g*-PEG-Biotin layer formed a ring-like structure within the undercut region of the hole mask, facilitating selective avidin binding. While the hole mask was maintained, the disk region in the middle of the PEG-Biotin ring was then backfilled by the deposition of PAcrAm-*g*-PEG-N_3_, a polymer capable of undergoing a copper-free SPAAC click chemistry reaction with DBCO-conjugated biomolecules. This strain promoted biorthogonal ligation of the DBCO group with the terminal azide group of the PEG polymers, which would form a stable triazole linkage between the surface and the biomolecule.

At this stage, we incorporated the sacrificial lift-off resist layer strategy detailed earlier to facilitate the concurrent deposition of two biospecific PEG brushes within each hole mask. Concurrently, the interstitial regions between the hole masks were treated with an antifouling PAcrAm-*g*-PMOXA polymer layer. The fabrication steps and surface analysis of nanopatterned biospecific polymers are delineated in Figures S6 and S7.

We examined the functionality of these biospecific tags via sequential incubation with SA and DBCO-conjugated bovine serum albumin (BSA), subsequently monitoring nanopattern formation. Figure 2c visualizes the resultant ring-like structures, characterized using fluorescently labeled SA, with the dark core corresponding to the original 500 nm nanoparticle employed in hole-mask construction.

Utilizing liquid AFM, we evaluated the surface topography pre- and postprotein incubation. Preincubation scans exhibited a nearly uniform surface, distinguished by a nominal 0.5 nm elevation in regions previously constituting the hole mask and hosting dual biospecific-tagged PEG brushes (Figure S8). After SA incubation, these ring-like regions displayed a height increase relative to the central disk area, which remained at the same level as the background surface, indicating specific binding of SA to the patterned PEG-Biotin regions. Super-resolution DNA-PAINT microscopy^51,52^ further confirmed the formation of these specific SA ring nanopatterns (Figure S9).

After SA incubation, samples were subjected to overnight incubation with fluorescently labeled DBCO-BSA and designed to react covalently with the predeposited PAcrAm-*g*-PEG-N_3_ layer residing in the former Cr disk positions within each hole mask. Previously, small molecules and peptides have been immobilized in this way, but this represents the first demonstration of direct immobilization of proteins at surfaces by SPAAC reactions.^31,32^ As predicted, fluorescence imaging revealed the specific assembly of DBCO-BSA in the central disk regions of the hole mask, as illustrated in red in Figure 2e. These were encircled by the ring-like arrangements of SA molecules, shown in green. Liquid-AFM data, shown in Figure 2f, confirmed these findings, showing a notable elevation in the central disk region postincubation with the DBCO-BSA, a region that was previously aligned with the background topography.

Having validated the directed assembly of proteins on subdiffraction limit structures (Figures S9–S11), we next investigated the feasibility of reversing the spatial arrangement of the deposited tags. Such an inversion could be advantageous for certain experimental conditions that necessitate alternate geometric orientations of the biomolecules without alterations to the biomolecular tags on the biomolecules themselves. Our findings, illustrated in Figure S12, confirm that such a rearrangement is attainable. However, as we have outlined in the previous work, the fabrication sequence proposed in Figure S6 remains optimal.^41^

Beyond modulation of the size of the nanoparticle-defined “disk region” and the hole mask, the surrounding “ring region” dimensions can also be precisely controlled. The minimum diameter of this ring is constrained by the size of the nanoparticle and the hole mask, while the extent of the generated undercut dictates the maximum diameter. We manipulated the dimensions of this region by varying the duration of O_2_ RIE. Structural modifications were assiduously monitored using SEM and fluorescence microscopy (Figures S13 and S14).

Figure S15 reveals a linear relationship between the etching time and the diameter of the undercut region. For 510 nm hole masks, we determined that the diameter of the undercut could be expanded from 600 to 950 nm by doubling the etching duration, yielding a rate of 20 nm per minute for our experimental conditions. Should larger undercut dimensions be desirable, increased particle spacing would be required to preclude the convergence of undercut regions and potential destabilization of the sacrificial PMGI layer. As a potential alternative, tetramethylammonium hydroxide-based developers could be employed as an alternative to the O_2_ RIE for isotropic etching of the PMGI layer within the hole masks.

In addition to controlling the undercut region, the shape of the metallic structure, currently forming the disk region, could also be changed. Angled metal evaporation into the hole mask has already been demonstrated to form structures such as crescents, bars, and hooks.^53−55^ While requiring reoptimizing the presented protocol’s parameters, such as the metal etching time, protein patterning of these structures rather than only the disk structures presented in this paper should be feasible.

### Triligand Nanopatterning: Extending the Surface Click Chemistry Toolbox

Expanding upon the arrangement of two biospecific tags at the nanometer scale using the HCL technique, we contemplated the feasibility of adding a third distinct biorthogonal tag to the interstitial areas between the nanostructures. Initial experiments focused on the potential for nonspecific protein adsorption to these background regions. Contrary to employing the antifouling PAcrAm-*g*-PMOXA polymer as a background layer, we directly deposited a protein layer that would adhere nonspecifically to these regions while being excluded from the nanostructured domains due to the absence of corresponding biospecific tags.

While this approach yielded some success, a notable drawback emerged: the nonspecifically adhered proteins were susceptible to displacement by subsequently deposited biospecific tagged proteins. Therefore, we observed significant off-target binding of the biospecific proteins to the background region (Figure S17).

To address the observed shortcomings of nonspecific binding in the interstitial regions, we investigated substituting the antifouling PAcrAm-*g*-PMOXA polymer with an orthogonally functionalized PEG brush holding a third biospecific tag. Among the candidates, we evaluated nitrilotriacetic acid (NTA) as a biospecific tag to capture His-tagged proteins, a strategy previously executed on PEGylated substrates.^35^

While the NTA-functionalized PEG brush demonstrated a high affinity for 9-His tagged proteins, it suffered from a high dissociation rate. Specifically, more than 50% of the adsorbed protein layer was desorbed within 2 h postincubation (Figure S16). Moreover, the NTA modification did not fully uphold the desired antifouling properties; we observed a significant level of nonspecific interactions with proteins, compromising the antifouling efficacy to below 90%. Thus, while the NTA-functionalized PEG brush offers a potential way to introduce a third biospecific ligand, the observed high dissociation rate and suboptimal antifouling performance necessitate alternative strategies.

Considering the limitations of NTA-functionalized PEG brushes, we shifted our focus toward next-generation click chemistry methods, specifically, the inverse electron-demand Diels–Alder (IEDDA) reaction. The biorthogonal nature of IEDDA, coupling an electron-rich diene such as tetrazine (TZ) with an electron-poor dienophile such as trans-cyclooctene (TCO), offers a reaction rate that is orders of magnitude faster than the SPAAC reaction, with reported kinetics reaching up to 10^3^ M^–1^ s^–1^ compared to 10^–1^ M^–1^ s^–1^ for SPAAC in physiological buffers, without the need for auxiliary reagents. This irreversible reaction between the TZ and TCO reaction pair forms a dihydropyridazine bond and releases the inert N_2_ gas, the only side product during the reaction.^56,57^

To explore the utility of these IEDDA reagents in our context, we synthesized PEG-TCO and PEG-TZ brushes. Although IEDDA reaction kinetics is well documented, the covalent immobilization of biomolecules onto fully PEGylated surfaces using IEDDA has not been well studied. This motivated their characterization, focusing on their antifouling attributes and reactivity toward TZ- and TCO-tagged biomolecules.

**Figure 3:**
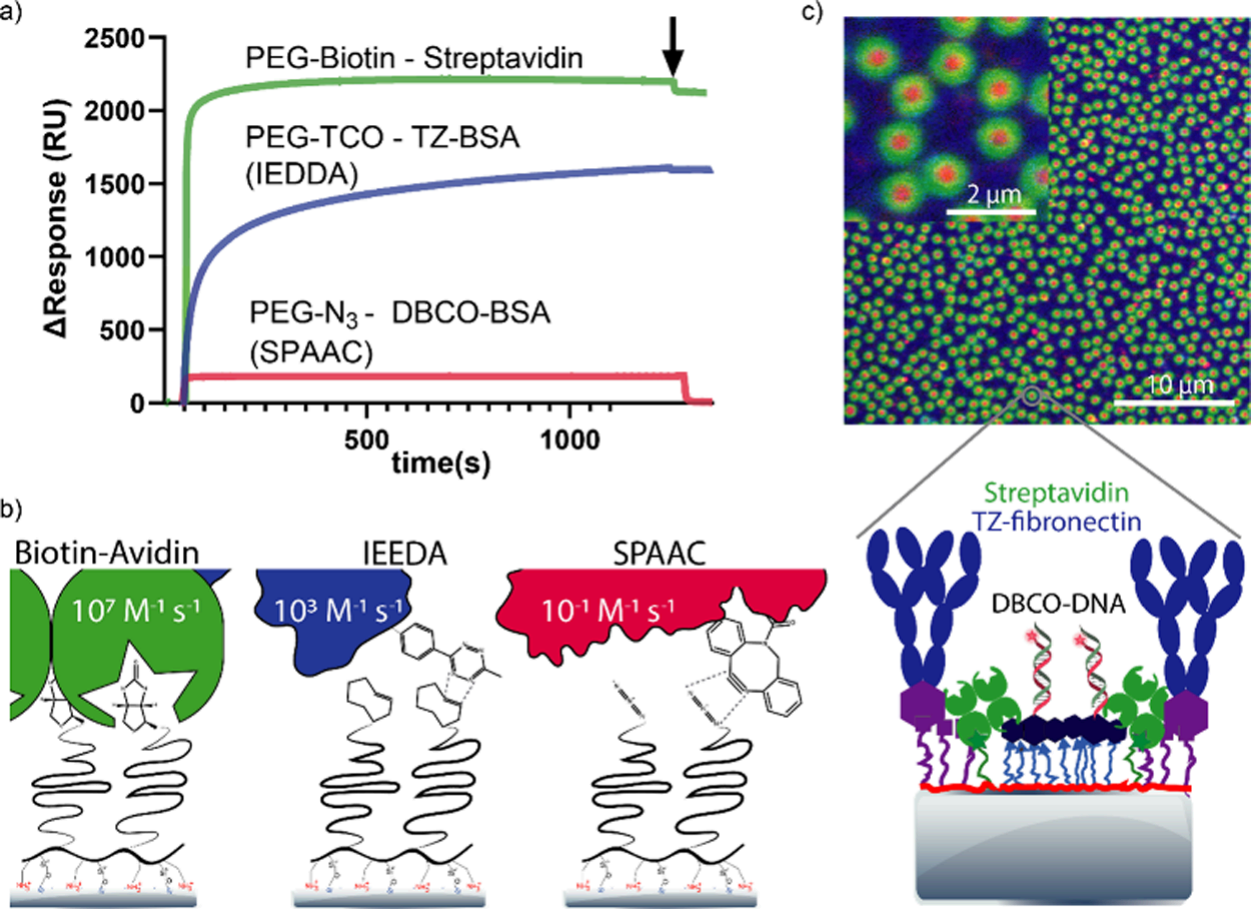
Comparison of different biospecific tag reactivities. (a) SPR sensorgram showing binding of targets at the same concentration to different biospecific PEG brushes. The arrow indicates the end of the injection. (b) Reactions and the reported second-order reaction rates of the reactions. The second-order reaction rates are reported from refs (56−58). (c) Fluorescence image of the triligand 800 nm protein nanopattern: green streptavidin, red DBCO-BSA, and blue tetrazine-labeled fibronectin.

Employing a SiO_2_-coated SPR chip to mimic our patterned substrate’s background region, we studied the antifouling and reactivity profiles of the 3.4 kDa PEG brushes functionalized with TZ and TCO. In a comparative analysis, PEG-TCO exhibited marginally superior antifouling characteristics, inspiring us to select this tag for subsequent experimental phases. Remarkably, both TZ and TCO demonstrated rapid reaction kinetics on the surface, nearly replicating the well-established biotin–avidin interaction, with saturation levels reached within a 20 min time frame (Figure 3a). This performance starkly contrasts with the slow binding dynamics observed in DBCO–N_3_ SPAAC reactions, where even after a 20 min exposure, minimal DBCO-BSA binding was detected, demonstrating the need for long (typically overnight) exposure for useful levels of coupling.

The temporal dynamics of these bioorthogonal reactions provide keen insights into their suitability for various applications (Figure 3b). For the biotin–avidin reaction, the rapid kinetics make it a matter of seconds to minutes for completion within the utilized protein concentration range (25–100 μg/mL ≈ 0.5–1.5 μM). On the other hand, the IEDDA reaction takes minutes to hours, and the SPAAC reaction extends from hours to days. These reaction speeds can be modulated by elevating the reaction temperature, which exponentially increases reaction rates due to increased collision frequency and diffusion rates.^59^

However, it is essential to underscore that heightened reactivity often comes at the expense of stability. Our prior work showed that biotin-tagged molecules manifest greater stability than their N_3_-tagged counterparts under comparable fabrication conditions.^41^ Further complicating matters, we found that PEG-TCO is notably sensitive to the 75 °C heat and Cr etching steps employed in our fabrication process, effectively limiting its application to the background region of the substrate. Moreover, when subjected to ambient light, PEG-TCO suffers a marked drop in reactivity toward TZ, likely attributed to cis–trans photoisomerization into a less reactive trans conformation, as published in recent studies.^60^

By using PAcrAm-*g*-PEG-TCO as a replacement for the antifouling PAcrAm-*g*-PMOXA layer in the background region (Figure S6), we successfully orchestrated the orthogonal patterning of three distinct biomolecules in geometrically defined regions with nanometer-scale precision (Figure 3c).

Specifically, we demonstrated the covalent immobilization of TZ-labeled fibronectin (FN), a large molecule exceeding 450 kDa, into the PEG-TCO functionalized background. We achieved the covalent attachment of DBCO-conjugated DNA oligonucleotides onto the patterned PEG-N_3_ layer within the nanopatterned regions. This was advantageously encircled by SA bound to a PEG-Biotin functionalized area, providing a versatile platform for tethering additional biotinylated biomolecules in the so-called ring region.

This novel approach not only elevates the complexity of surface functionalization but also opens opportunities for sophisticated bionano interfaces, accommodating a multitude of biomolecular interactions in a highly controlled spatial configuration.

### Mechanical Effects of Nanoscale Proximal Patterning of Integrin-Ligand and Signaling Molecules

In a proof-of-concept study, we leveraged the precision and versatility of our nanopatterning system to investigate the significance of receptor proximity effects, a phenomenon with established relevance in cellular signaling cascades, including those involving epidermal growth factor receptor (EGFR) and integrin.^61,62^ EGFR signaling cascades are known to modulate various cellular behaviors, such as proliferation, focal adhesion assembly, and cellular morphology.^63^

We patterned substrates with adhesive dsDNA functionalized with cRGD, a peptide that serves as an integrin ligand. These were organized into 800 nm nanopatterns, an architecture we previously demonstrated to allow for the assembly of focal adhesion complexes.^6,64^ Adjacently, we engineered a ring region around each cRGD nanodisk, populating it with epidermal growth factor (EGF), to enable an induced proximity of EGFR and integrins.

Crucially, given the evidence for the significance of the biologically relevant orientation of EGF on surface activity as indicated by Liberelle et al., we chose to incorporate*Staphylococcus aureus* Protein A into the ring region.^65^ This Fc-binding protein facilitates the orientation of the chimeric Fc-fused EGF in its bioactive configuration.

**Figure 4:**
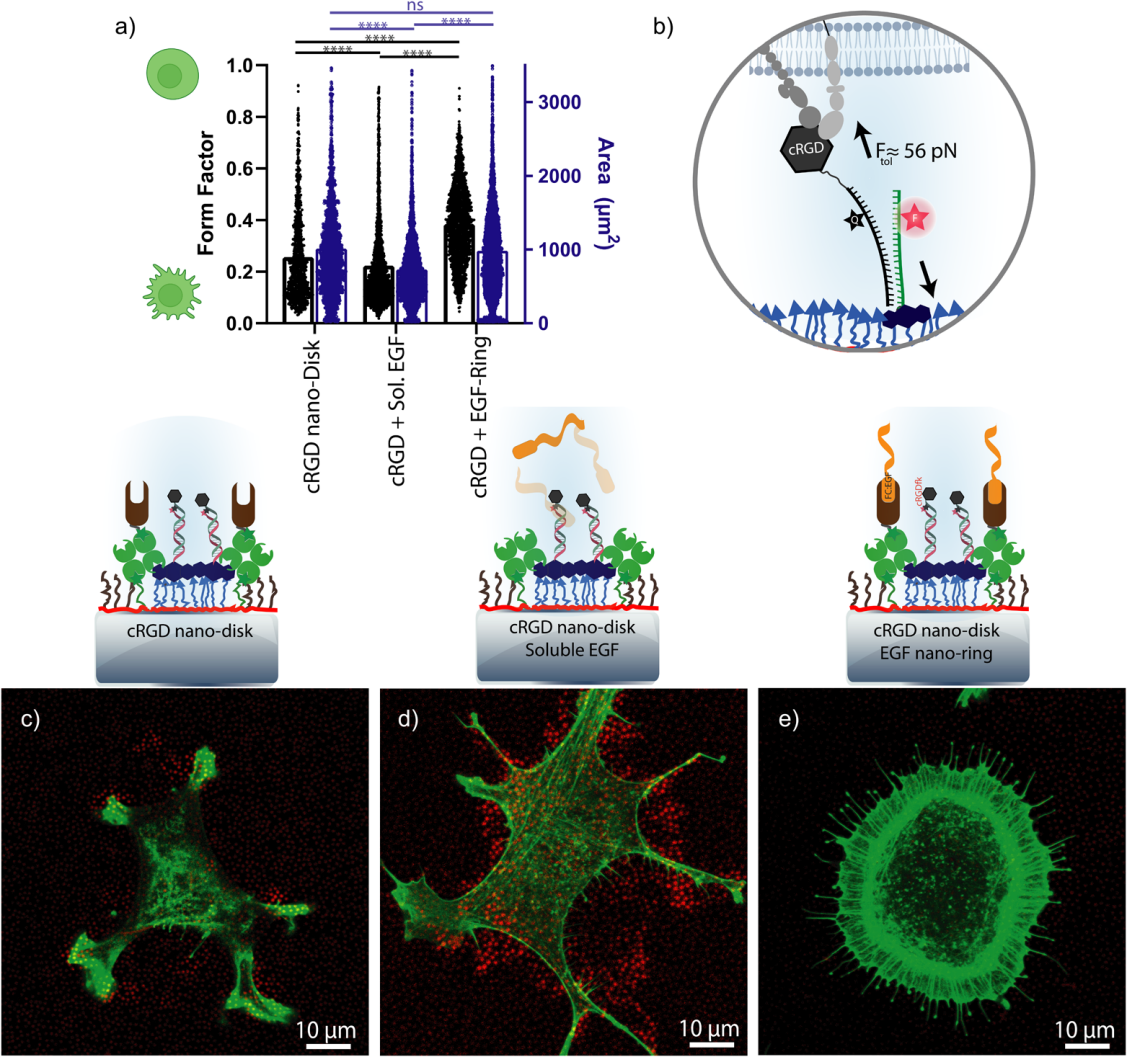
Proof-of-concept in vitro experiment with 3T3 fibroblasts and 800 nm patterned adhesive cRGD-disks and epidermal growth factor signaling rings. (a) Quantification of cell form factor and spread area on different patterns. Bars show the means. Black dots represent the measured form factor of individual cells, while blue dots represent their area. Statistical analysis is performed by one-way ANOVA with Tukey’s multiple comparison, **** indicates *P* < 0.0001, ns indicates no significant statistical differences. Data were obtained from individual cells (*n* ≥ 2000) with at least two technical repeats and two independent biological replicates. (b) Schematic showing a ruptured turn-on DNA tension gauge tether upon the interaction of the cells with nanopatterned tension probes. (c–e) Representative fluorescence images of cellular F-actin (green) and mechanically opened 56 pN cRGD tension probes (red).

The population of cells attached to surfaces incorporating both RGD and EGF shows a clear change of cell shape compared to RGD alone or RGD presented with EGF in solution with a more round shape and cortical actin compared to a stellate shape. The quantifiable metrics of cell behavior offer significant insights into the efficacy of our nanopatterning strategy and the ability for large-area patterning. Figure 4a presents data on the spread area and form factor (4·π·area/perimeter^2^) of 3T3 fibroblasts after a 1 h incubation on the nanopatterned substrates. Control substrates lacking surface-bound EGF or featuring EGF covering the background region between the 800 nm cRGD disks as well as nanopatterned EGF substrates without the cRGD were also prepared for comprehensive analysis (Figures S18 and S19). Additionally, a condition with nonsurface bound, soluble EGF-Fc at a concentration of 1.5 nM was examined.

Notably, the presence of surface-bound EGF in close proximity to the cRGD disks led to a marked increase in the form factor of the cells compared to either the absence of EGF or its soluble form, corresponding to a more rounded morphology. Furthermore, upon engagement of the cells with surface-bound EGF either as nanorings in the proximity of the cRGD disks or covering the whole background region around the cRGD nanodisks, the aspect ratio was decreased (Figure S18). These indicate that surface-bound EGF results in an overall more rounded cell shape with increased cell membrane ruffling, potentially due to the altered focal adhesion assembly upon EGF interaction.^63^

This observation fits prior studies indicating that surface-bound EGF elicits a more robust cellular response than even saturating concentrations of soluble EGF.^66,67^ These results collectively demonstrate that the nanoscale patterning of biologically active EGF in the vicinity of the integrin-binding cRGD motif is feasible and functionally impactful.

The most round cells were observed when the adhesive cRGD peptide was emitted from the surface. Without the presence of either cRGD or EGF on the surface, the nonfouling surfaces showed negligible cell binding, as previously reported for similar nanopatterned surfaces.^41^ Nanopatterning of EGF on the surface allowed the cells to adhere to the surface; however, these cells showed, on average, more than a 50% decrease in the spread area and aspect ratio compared to those in the presence of cRGD, indicating a low degree of interaction with the surface (Figure S18).

The force dynamics at focal adhesions provide a crucial axis for understanding cellular behaviors, such as migration and mechanotransduction. Leveraging cRGD-functionalized dsDNA nanodisks, designed as DNA tension gauge tether (TGT) molecular tension probes,^68^ we aimed to explore the influence of EGFR-integrin crosstalk on integrin-generated forces. These TGTs are designed in a shearing configuration to undergo irreversible duplex rupture when mechanical forces surpass the 56 pN threshold. The rupture results in decoupling a quencher from its adjacent fluorophore, thereby leading to a detectable increase in fluorescence, as illustrated in Figure 4b.

In the absence of EGF, 3T3 fibroblasts showed an archetypal spindle shape replete with irregular cytoplasmic protrusions (Figure 4c). These protrusions were sites of noticeable mechanical activity, as evidenced by the rupture of several cRGD-functionalized TGTs localized within the predefined nanopatterned domains following a 1 h incubation on the engineered surface.

Intriguingly, the solubilized form of EGF elicited an alternative mechanical response; corroborating prior observations by Rao et al., we recorded a significant augmentation in force generation, manifested as a series of ruptured TGTs localized within proximal nanopatterns to the cellular extensions (Figure 4d).^69^

Interestingly, the copatterning of EGF alongside cRGD motifs led to an unexpected decrease in mechanical events exceeding 56 pN, as evidenced by sparse instances of fluorescence signals from the ruptured nanopatterned TGTs (Figures 4e and S18). The cell shape was, however, consistent with prior observations of an increased measured form factor and elongated filopodia extending toward the cRGD-functionalized regions. Our data indicate that the nanoscale presentation of EGF at surfaces in proximity to cellular adhesions may give altered signaling compared to presentation away from cellular adhesions.

## Conclusions

In summary, we have introduced a robust method for the nanoscale arrangement of up to three distinct ligands on fully passivated PEG brush surfaces over large areas. Utilizing a modified HCL protocol, we engineered apertures for site-specific deposition of PEG brushes with three biorthogonal tags including two copper-free covalent click chemistry tags. This technique allows for the controlled placement of biospecifically tagged PEG brushes in defined geometric locales by strategically removing the physical barriers. The fully transparent and topography-free substrates can be assembled onto standard format microwell plates for imaging and analysis and are compatible with all established fluorescence-based in vitro protocols.

In a proof-of-concept experiment, we effectively validated the engineered surfaces by quantifying the impact of proximally patterned EGF and cRGD on the cellular morphology and integrin-mediated molecular tension in 3T3 fibroblasts. This is the first time that cellular forces have been measured using nanopatterned DNA-based tension probes.

The framework facilitates future studies of subcellular interactions of multiple ligands with control over ligand positioning at the nanoscale. These are not limited to clustering effects of signaling/adhesive and stimulatory/inhibitory molecules. Additionally, this platform provides a stable and sophisticated in vitro culture with the potential to mimic a more native-like environment for molecular force measurements.

## Experimental Methods

### Random Cograft PEG Brushes

Poly(acrylamide)-*g*-(PMOXA, 1,6-hexanediamine, 3-aminopropyldimethylethoxysilane)) PAcrAm-*g*-PMOXA (NH_2_,Si), poly(acrylamide)-*g*-(PEG-N3, 1,6-hexanediamine, 3-aminopropyldimethylethoxysilane) PAcrAm-*g*-PEG-N3(NH_2_,Si), poly(acrylamide)-*g*-(PEG-N3, 1,6-hexanediamine, nitrodopamine) PAcrAm-*g*-PEG-N3(NH_2_,ND), and PLL-*g*-PEG-NTA(50%) were purchased from SuSoS, Switzerland.

To synthesize the remaining biospecific PEG brushes, the PAcrAm-*g*-PEG-N_3_ polymer’s terminal azide (N_3_) group was modified with a bifunctional DBCO-PEG4-Biotin/Tetrazine/TCO (Click Chemistry Tools). The PEG-N_3_ polymer was dissolved in 1 mg/mL 1 mM (4-(2-hydroxyethyl)-1-piperazineethanesulfonic acid) (HEPES) buffer set to pH 7.4 and reacted with an excess DBCO modifier overnight at room temperature with shaking in the dark. To remove the excess DBCO modifier, the polymer was spin-filtered 5 times in an Amicon centrifugal filter with a 30 kDa cutoff.

### Protein Labeling

Bovine serum albumin, streptavidin, fibronectin, and staphylococcal Protein A were purchased from SigmaAldrich. To label these proteins, *N*-hydroxysuccinimide (NHS) esters with different functional groups, such as DBCO, TCO, biotin, or TZ, have been used in 8 times molar excess to react with proteins and ligands of interest primary NH_2_ groups. Dual labeling of the same protein with a biospecific tag and fluorophore was performed by incubating the fluorophore NHS esters at the same time as described above. The reaction was performed in 50 mM HEPES buffer with 150 mM NaCl at pH 8.0 for 1 h at room temperature with shaking.

The labeled ligand/protein was buffer exchanged by using Amicon centrifugal filters with an appropriate molecular cutoff. This also allowed for the removal of excess NHS ester. The final protein concentration was determined by UV A280 measurements.

### Preparation of Nanopatterned Substrates

#1.5H coverslips or polished Si-wafers were cleaned by ultrasonication in acetone, and isopropanol alcohol was dried under a stream of N_2._ Immediately before use for the next fabrication step, the substrates were treated with 3 min-long oxygen plasma reactive ion etching (RIE) (Vision 300 MK II, Advanced Vacuum) with a radio frequency generator power of 100 W and 100 SCCM O_2_ flow at 25 mTorr pressure.

A modified version of hole-mask colloidal lithography (HCL) was adopted to create structures. A 170 nm layer of LOR 2A (KayakuAM) was spun-coated at 3000 rpm with an acceleration of 7000 rpm/s for 45 s on the clean substrate. The substrate was then baked for 2 min on a hot plate at 180 °C to evaporate the solvent. The polymeric surface was made hydrophilic by a short 10 s O_2_ RIE (25 W, 100 SCCM O_2_, 25 mTorr). Next, a modified protocol for sparse colloidal lithography was used to form a sparse monolayer of nanoparticles on the surface. Three polyelectrolyte layers were deposited on the substrates to achieve a net positive charge on the surface and stabilize the subsequent formation of the monolayer of the nanoparticles. First, polyethylenimine–branched *M*_w_ 25,000 (SigmaAldrich) at 2 wt %, followed by poly(sodium 4-styrenesulfonate) *M*_w_ 70,000 (SigmaAldrich) at 2 wt %, and finally poly(diallyldimethylammonium chloride) *M*_w_ 200,000–350,000 (SigmaAldrich) at 0.5 wt %. Each solution was incubated for 30 s on the surface, followed by rinsing with DI H_2_O for 30 s and drying under a stream of N_2_.

200–800 nm negatively charged sulfate latex beads (ThermoScientific) were deposited at 0.2 wt % (2 to 30 min) to form a sparse monolayer of nanoparticles. The measured colloidal particle dimensions and charge densities are provided in Table S1. The unbound particles were removed by rinsing with DI H_2_O. To avoid capillary force induced aggregation of the assembled particle monolayer, the substrates were dropped in boiling water for 1 min to increase the adhesion and then dried under a stream of N_2_. Next, a 20 nm-thick Ti-metallic mask resistant to reactive O_2_ plasma etching was deposited via e-beam thermal PVD (Cryofox Explorer 500 GLAD, Polyteknik, Denmark). The nanoparticles are tape-stripped away, leaving nanoholes in the plasma-resistant Ti film on the sacrificial LOR layer. The sacrificial LOR layer was removed with a strong O_2_ RIE (100 W, 40 SCCM O_2_, 25 mTorr) for 10–20 min to achieve an undercut underneath the hole mask. Cr (1 nm) was deposited via e-beam thermal PVD from 78 cm at a rate of 0.08 Å/s with 5 rpm rotation. After PVD deposition and subsequent short plasma RIE, the substrates were heated to 180 °C for 2 min to increase substrate-LOR resist adhesion. To form the antifouling layers, the substrate was incubated with 0.1 mg/mL PAcrAm-*g*-PEG-Biotin(NH_2_,Si) in 1 mM HEPES pH 7.4 for 30 min on parafilm. The Cr disk was etched away using a 0.2 μm filtered ceric ammonium nitrate-based chromium wet etchant (SigmaAldrich Catalog ID: 651826) for 30 s. After 30 min of incubation with 0.1 mg/mL PAcrAm-*g*-PEG-N_3_(NH_2_,Si), a monolayer of PEG-N_3_ brushes formed on the newly exposed SiO_2_ area underneath the Cr disk. The Ti hard mask and the LOR resist are entirely removed by sonication of the substrates in 75 °C preheated dimethyl sulfoxide (DMSO) for a total of 33 min in 4 different acidic Piranha-cleaned glass beakers (Warning: Piranha solution is EXTREMELY corrosive and potentially explosive!!!). The substrates were then rinsed with DI H_2_O and dried under a stream of N_2_. The exposed background was then either incubated directly with the protein of interest or first incubated with PAcrAm-*g*-PEG-TCO (NH_2_,Si) at 0.1 mg/mL for 30 min before incubation with the labeled proteins. Subsequently, the patterned biomolecules were incubated in PBS. Streptavidin was incubated for 30 min at 50 μg/mL. Biotinylated Protein-A was incubated at 10 μg/mL for at least 2 h. DBCO-labeled proteins were incubated at approximately 1 μM (100 μg/mL) overnight at 37 °C. DBCO-labeled DNA was incubated under similar conditions at 250 nM. Tetrazine-labeled proteins were incubated at the same concentration at room temperature for 1 h.

### Surface Characterizations

An FEI Magellan 400 SEM was used to acquire images based on secondary electron detection. The presented SEM images were acquired with an incoming electron beam of 5 kV and a nominal beam current of 50 pA.

A Zeiss LSM 700 confocal laser scanning microscope (CLSM) with oil immersion (*n* = 1.518) 64× and 100× objectives with an aperture of 1 airy unit was used to image the protein nanopatterns. Lasers were set to 2%, and no digital offset was applied. The images were contrast-corrected and exported by FIJI.

Liquid AFM scans were done using a Bruker Multimode 8 with a liquid cell and ScanAnalyst-Fluid+ tips with a nominal radius of 2 nm. PeakForce Quantitative Nanomechanics Mode with a set point under 1 nN was used for measuring the protein pattern structures in PBS. Tapping mode AFM scans in air were done using Bruker Dimension Edge and RTESP-300 tips with a nominal radius of 8 nm. All data were plane-fitted through three points, and lines were aligned by modulus in Gwyddion before analysis or presentation.

XPS data acquisition was performed using a Kratos Axis UltraDLD instrument (Kratos Analytical) equipped with a monochromated Al kα X-ray source operating at 10 kV and 15 mA (150 W). Survey spectra (binding energy (BE) range of 0–1100 eV with a pass energy of 160 eV) and high-resolution spectra (with a pass energy of 20 eV) were obtained to determine the presence of an element on the surface. All data presented are based on an average of 3 scans of a single spot. The acquired data were analyzed by CASA XPS. The BE scales for the high-resolution spectra were calibrated by setting the BE of the C 1s C–C/C–H component to 285.0 eV. Peak fittings were performed using Shirley’s background and convolution of a Lorentzian with a Gaussian.

### Surface Plasmon Resonance (SPR)

Biacore SIA Au chips were purchased from Cytiva. The chips were sputter-coated with 4 nm of Ti, followed by 30 nm of SiO_2_ to resemble the SiO_2_/glass substrate surface chemistry used. Buffers and solutions were degassed by sonication and filtered with a 0.22 μm syringe filter prior to use. A Biacore 3000 was used for the measurements. All experiments were performed with a flow rate of 5 μL/min at 25 °C with concentrations similar to static conditions.

### DNA PAINT Super-Resolution Imaging

An Oxford Nanoimager S instrument in TIRF mode was used for DNA-PAINT super-resolution imaging. DBCO-terminated 7xR3 DNA docking strand (IDT, USA) was conjugated to the azide-modified streptavidin and purified using a 30 kDa MWCO Amicon Ultra centrifuge filter. A Cy3B-conjugated R3–7 nt imager was used at a concentration of 50 pM in imaging buffer C+ (PBS with the addition of 500 mM NaCl and 0.05% Tween-20) with the addition of an oxygen scavenger and triplet-state quencher (1× PCA, 1× PCD and 1× trolox). A 561 nm laser with a power density of ∼2.5 kW/cm^2^ was used to illuminate the sample, and 10.000–30.000 frames with an exposure time of 100 ms were acquired. The images were drift-corrected using 90 nm Au fiducial markers and reconstructed in Picasso.

### Cell Experiment with the cRGD Tension Probes

3T3-J2 fibroblast cells were cultured in Dulbecco’s modified Eagle’s medium (high-glucose DMEM with GlutaMAX, Thermo Fisher Scientific). Cells were incubated at 37 °C with 5% CO_2_ and supplemented with 10% (v/v) fetal bovine serum (FBS), 100 mg mL^–1^ penicillin, and 100 mg mL^–1^ streptomycin. The cells were passaged by trypsinization when they reached approximately 80% confluence. The DNA-based tension gauge tethers in shearing confirmation with sequence surface: 5′ GTG TCG TCG CT/iCy3/ATA CAT CTA 3′/3AmMO/ (IDT, USA) were functionalized with DBCO-NHS, and the ligand: 5′ TAG ATG TA[BHQ2-dT]GAG GCA CGA CAC 3′ [AmC3] (Eurofins Genomics, Germany)) strand first with DBCO-NHS and then with cyclo-RGD-Azide (Vivitide, RGD-3749-PI) and purified by RP-HPLC on a Phenomenex Evo C18 reverse-phase column using a MeCN gradient starting from 5% MeCN, 5% triethylammonium acetate (TEAA), to 95% MeCN over 35 min followed by 5 min with 95% MeCN. The tension probes were thermally annealed at 400 nM by heating to 95 °C and cooling to 25 °C during 25 min in 1× PBS with 10% molar excess of the ligand strand. The nanopatterned Ø25 coverslips were mounted to a Greiner 96-well bottomless plate (ID: 655000) using CO_2_ laser-cut double-sided medical pressure-sensitive adhesives tested for in vitro cytotoxicity according to ISO 10993-5 purchased from AdhesiveResearch, Inc. (ARcare 90106NB) as described previously.^41^ The nanopatterned substrates were blocked with 0.3 mg/mL BSA for 15 min before incubation with streptavidin and biotinylated Protein A, as described before. The annealed tension probes were incubated on the substrates overnight at room temperature at 250 nM. Next, EGF-Fc (GenScript, Z03377) used in the receptor proximity nanopatterning experiment was incubated for at least 2 h in 10 μg/mL for binding to Protein A. The same protein was used at 1.5 nM for the soluble EGF experiment. The cell experiments were performed in the same medium used for culturing, excluding FBS at a seeding density of 3000 cells/cm^2^. The cells were fixed for 10 min in 4% PFA and permeabilized in 0.2% Triton-X 100 for 10 min before staining for nuclei with 300 nM DAPI (Sigma-Aldrich) and F-actin with 50 nM Atto-488 Phalloidin (AttoTec, Germany) for 30 min. The cells were then immediately imaged using the high-content imaging system ImageXpress Pico (Molecular Devices) with a 20× objective. The cell experiments were repeated independently twice, with each independent repeat having at least two experimental repeats per condition. The results were then analyzed in CellProfiler^70^ using fluorescence intensity thresholding of the DAPI signal, and cells were segmented using probability masks generated using a trained machine learning pixel classification model in Ilastik. The tension probe images presented are based on CLSM images after background subtraction in Fiji.

### Statistical Analysis

The statistical analysis was performed using GraphPad Prism 8.3.0. The data were analyzed with one-way ANOVA followed by Tukey’s multiple comparisons test. *P* < 0.05 indicated statistical significance.

## Supporting Information Available

Additional information and characterization of surface preparation, super-resolution imaging, and extended cell experiment data (PDF)
