# Supplementary material for: Smart Biointerfaces via Click Chemistry-Enabled Nanopatterning of Multiple Bioligands and DNA Force Sensors
Source: ACS Appl Mater Interfaces. 2024 Apr 18;16(17):21534–45. doi: 10.1021/acsami.4c00831 (PMC11073048; doi:10.1021/acsami.4c00831)
Supplement: Supplementary file 1 — am4c00831_si_001.pdf [file am4c00831_si_001.pdf]

Supporting Information

Smart Biointerfaces *via* Click Chemistry-Enabled Nanopatterning of Multiple Bioligands and DNA Force Sensors

Ali Shahrokhtash[1,2], Duncan S. Sutherland[1,2*]

1) Interdisciplinary Nanoscience Center, Aarhus University, Gustav Wieds Vej 14, 8000, Aarhus C, Denmark
2) The Centre for Cellular Signal Patterns (CellPAT), Gustav Wieds Vej 14, 8000 Aarhus C, Denmark

[*]Corresponding Author: Duncan S. Sutherland

duncan@inano.au.dk

Table of Contents

Figure S1: SEM Side View of Tape-Stripped Hole-Masks and Partially Covered Nanoparticles

Figure S2: SEM Top View of Hole-Mask Post Tape Stripping

Figure S3: XPS Surveys and Quantifications of PMGI Resist Lift-Off vs Plasma Cleaned SiO_2

Figure S4: SEM Imaging of biotinylated Au Nanoparticles Binding to Streptavidin Patterns

Figure S5: SPR Study on the Impact of Cr Etching on PAcrAm-g-PEG-Biotin Layer Binding Efficiency

Figure S6: Stepwise Schematic of Three-Way Protein Nanopattern Fabrication

Figure S7 AFM Scans of the Surfaces During Different Stages of Fabrication.

Figure S8: Liquid AFM Scans of 200 nm Patterned Surface Pre-Protein Incubation

Figure S9: DNA-PAINT Super-Resolution Imaging of 200 nm Streptavidin Rings

Figure S10: Fluorescence Imaging of Dual Ligand Protein Patterns on 210 nm Nanostructures (Zoom-In)

Figure S11: Fluorescence Imaging of Dual Ligand Protein Patterns on 210 nm Nanostructures (Overview)

Figure S12: Effects of Biospecific PEG-Biotin and PEG-N3 Polymer Deposition Order

Figure S13: SEM Analysis of O2 RIE Etching Time on Nanoparticle Ring-Region Dimension

Figure S14: Nano-Ring Formation of Streptavidin on 500 nm Structures with Variable Etching Durations

Figure S15: Correlation of Etching Time and Diameter of Nanoparticle Ring Region

Figure S16: SPR Sensorgram of Rapid Desorption of 9-his GFP from PLL-g-PEG-NTA Surfaces

Figure S17: Tri-Ligand Patterning: Nonspecific Binding Analysis Without Anti-Fouling Polymer

Figure S18: 3T3 Fibroblasts on cRGD TGTs with EGF Control Experiment Quantification

Figure S19: Fluorescence Images of Cells on Control Surfaces

Table S1: Colloidal particles' specifications

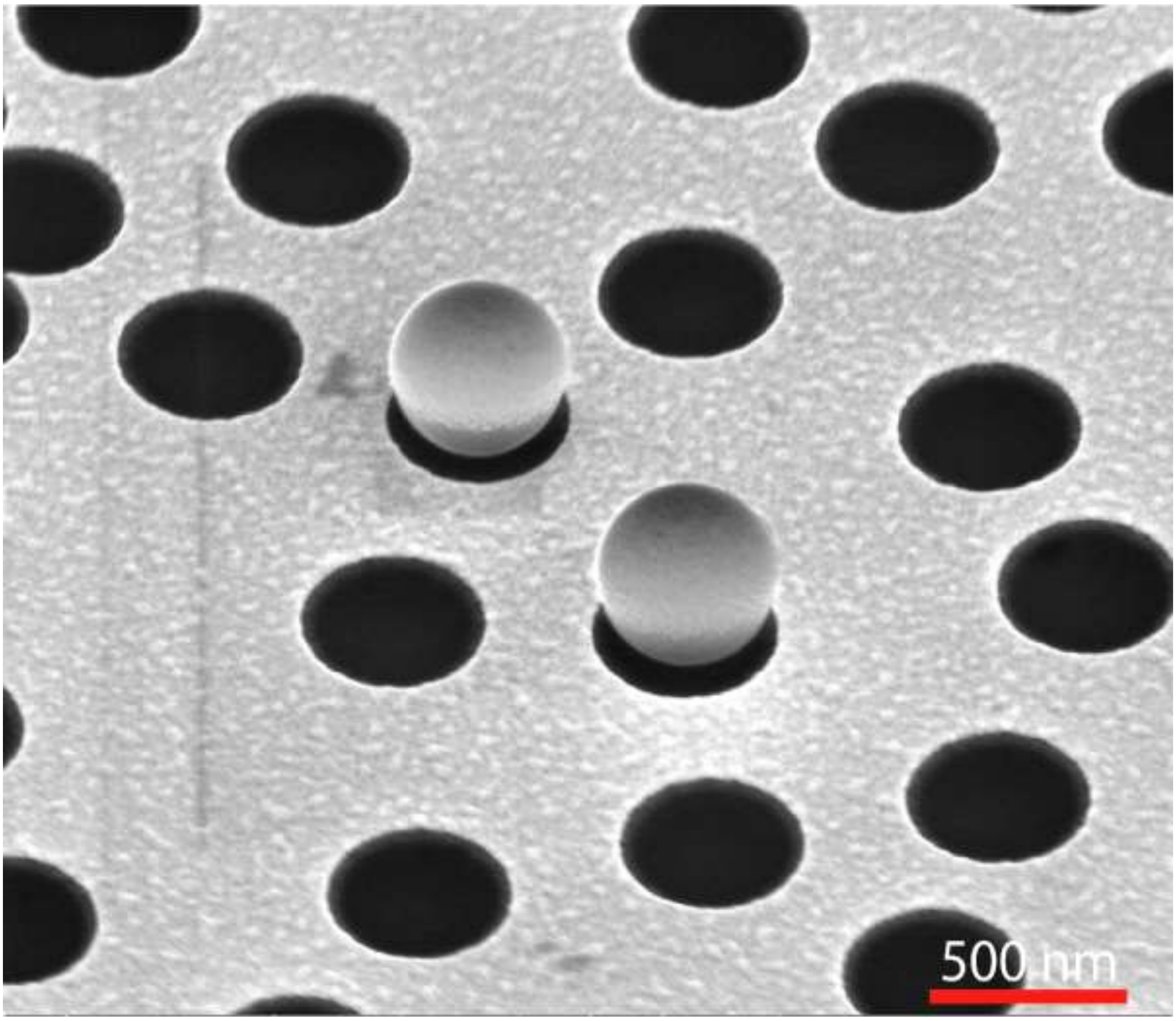

Figure S1 SEM side view of hole-masks generated after tape stripping a hole-mask and a couple of nanoparticles partially covered partially with Ti on the surface.

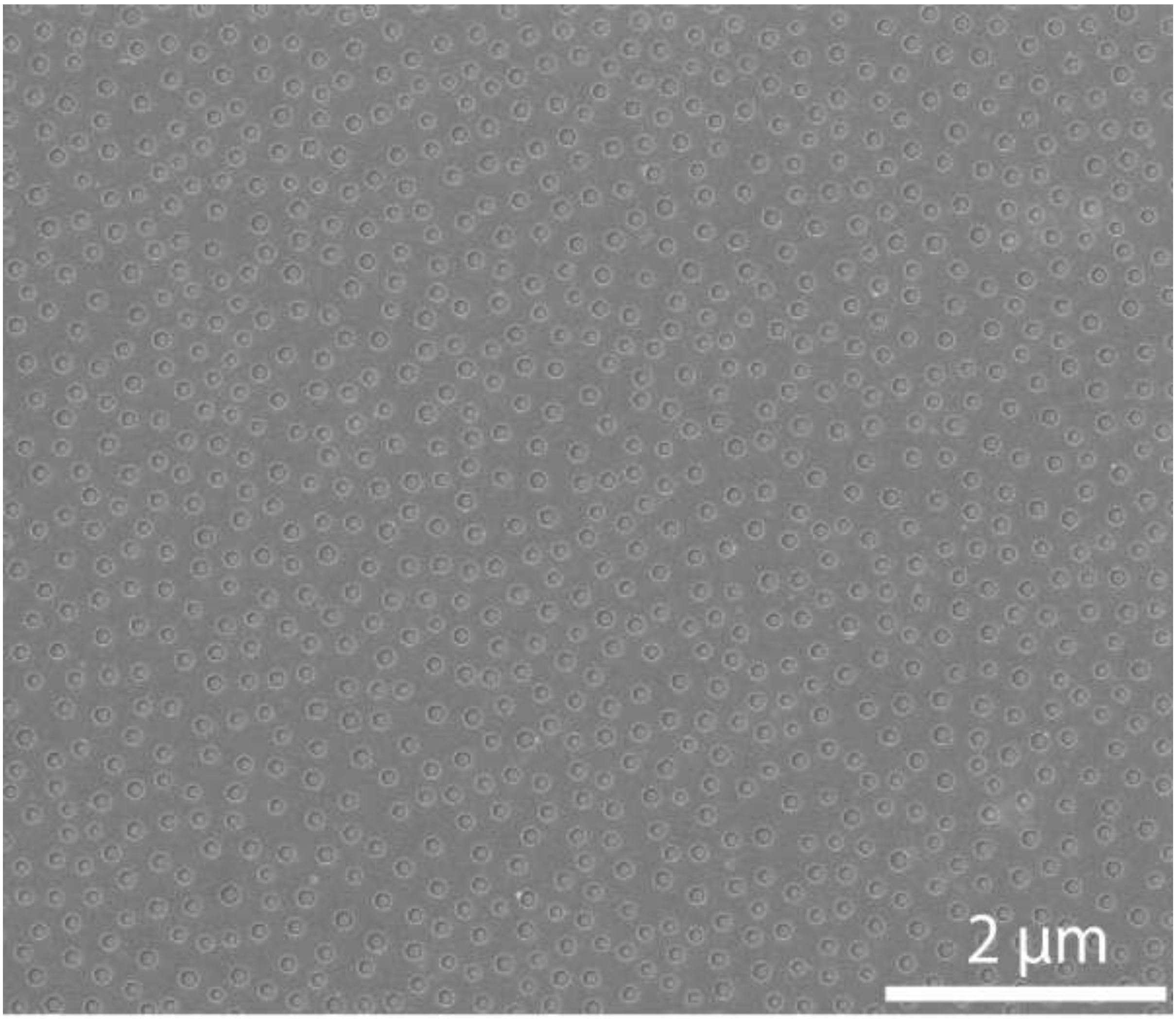

Figure S2 SEM top view of hole-mask after tape stripping.

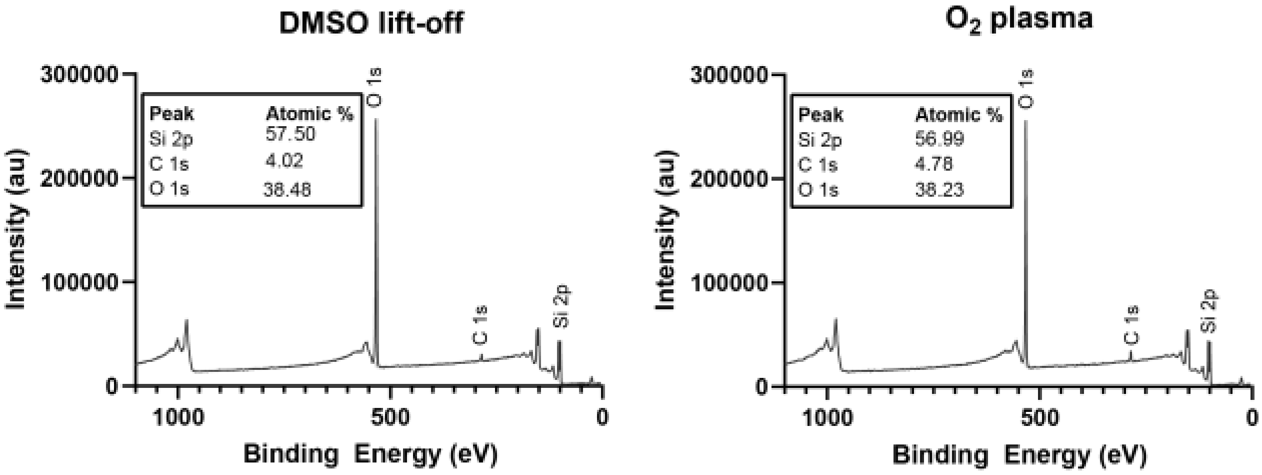

Figure S3 XPS surveys and quantifications after lift-off of the sacrificial PMGI resist in DMSO compared to O_2 plasma cleaned SiO_2 surface.

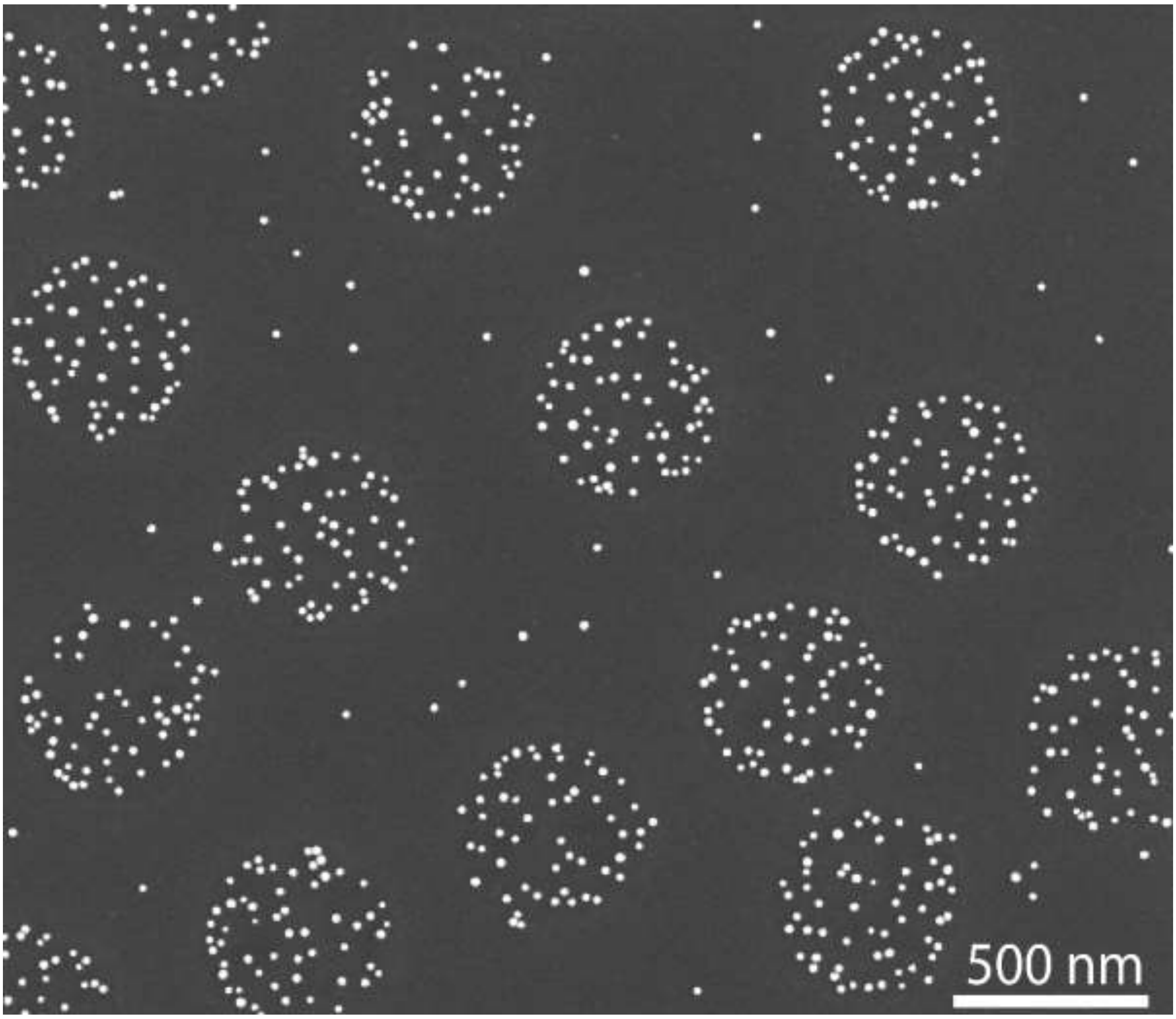

Figure S4 SEM image of 40 nm Biotin Au nanoparticles binding to 500 nm streptavidin nanopatterns

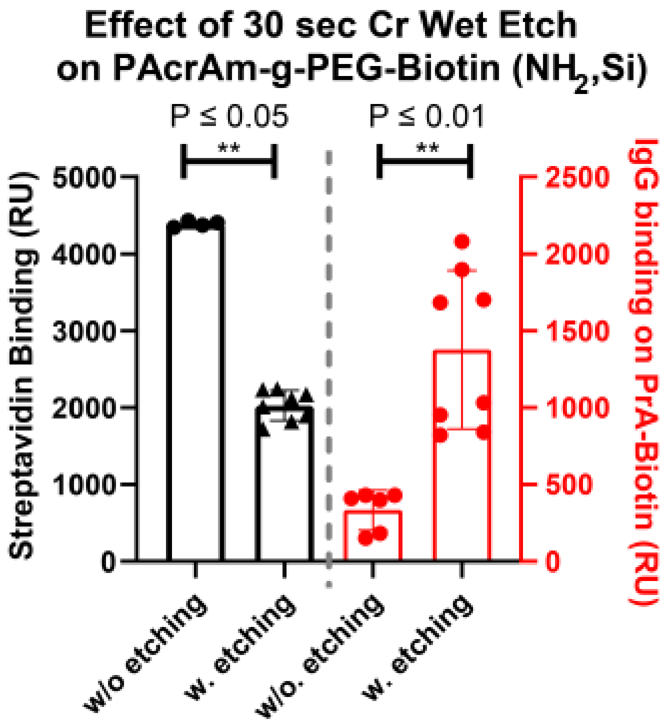

Figure S5 Summary of SPR experiments to study the effect of the short Cr etching step on the first deposited PAcrAm-g-PEG-Biotin layer. While this layer binds less streptavidin, probably due to the loss of some of the biotin tags on the surface, much more specific binding Fc-bearing protein through biotinylated Protein A is observed.

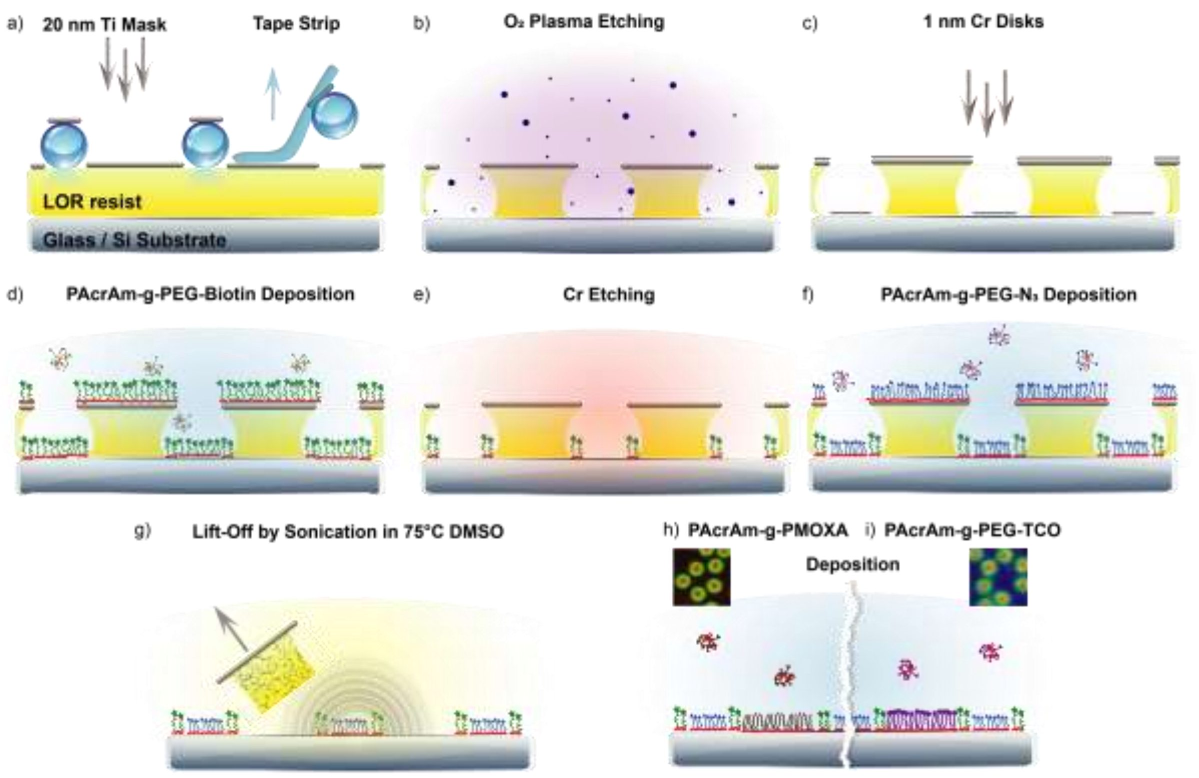

Figure S6 Schematic of 3-way fabrication protein nanopatterns fabrication. a) a hole-mask using a modified HCL protocol was produced. b) Using O_2 RIE, the sacrificial LOR layer underneath the particles, which is not protected by the 20 nm Ti mask, is etched away. c) A 1 nm Cr layer is deposited using thermal e-beam PVD with a deposition rate of 0.1 Å/s. D) PAcrAm-g-PEG- Biotin(NH_2, Si) is deposited. The PEG brushes cover the undercut with access to the substrate as well as the Cr disk and mask. e) 30 seconds of Cr wet-etching removes the Cr disks and strips the deposited PEG-Biotin on the disks away. This leaves patches with the same diameter as the Cr disks that PEG no longer covers. f) Deposition of PAcrAm-g-PEG-N_3 covers the newly exposed areas under the disks. g) The LOR layer is removed by sonication-assisted lifting-off for approximately 30 min in 75°C DMSO, and the background is revealed. h) The background region can be covered by the anti-fouling or i) biospecific PAcrAm-g-PEG- TCO.

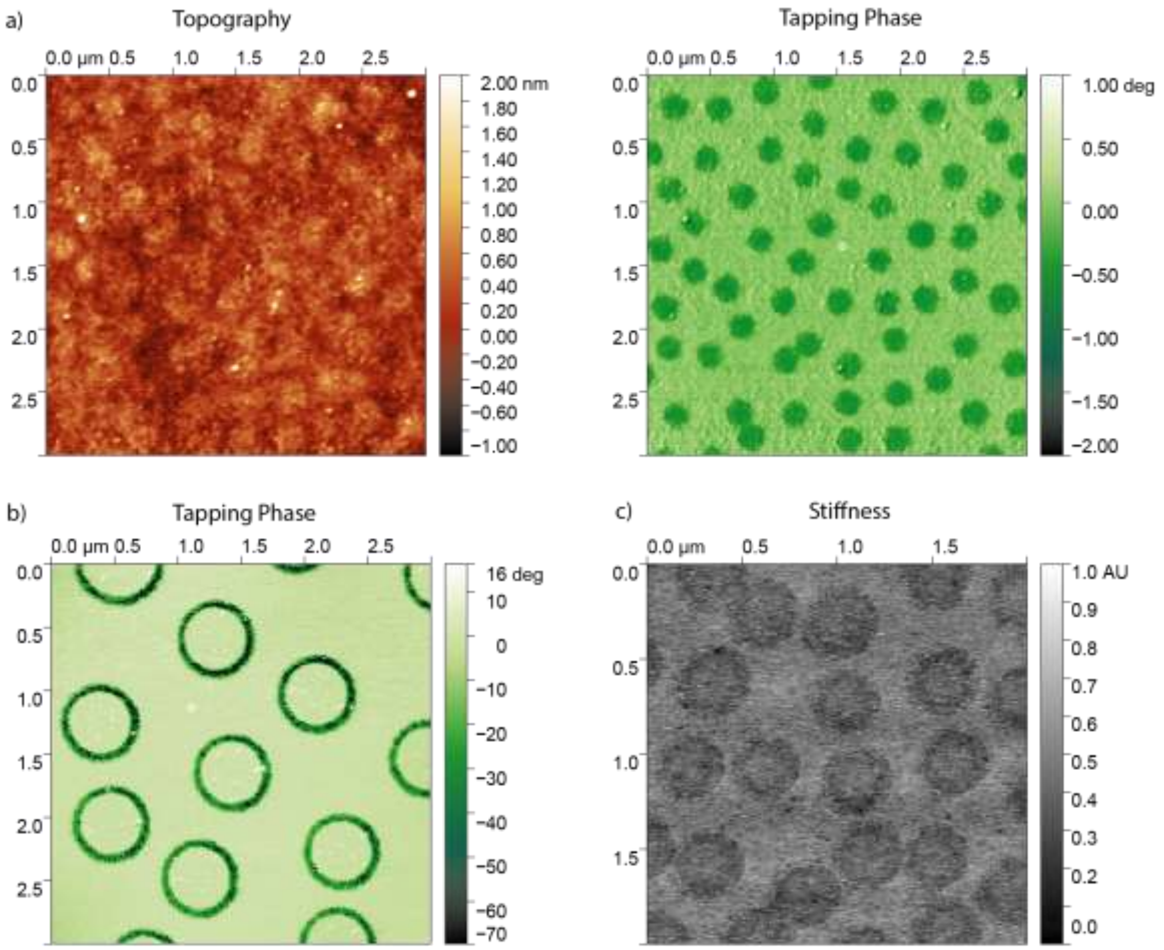

Figure S7 AFM scans of the surfaces during different stages of fabrication and deposition of the biospecifically tagged PEG-brushes. A) Tapping mode in air AFM topography and tapping phase scans of 200 nm patterned substrate after deposition of the PAcrAM-g-PEG-Biotin into the hole-mask, lift-off of the PMGI layer used to create the hole-mask and backfilling the background region between the nanoparticles with PAcrAM-g-PMOXA. The surface shows a planar substrate with very limited topographical features (z-scale 3 nm). Although the surface is planar, the biospecifically tagged PEG-biotin shows a different phase contrast than the PMOXA-coated background region. Circular structures – approximately 200 nm in diameter, corresponding to the size of the nanoparticles used to generate the patterns are clearly evident. b) tapping mode in air phase image of the PAcrAM-g-PEG-Biotin molecule deposited in the ring region around the nanoparticles. The AFM scan is done after etching the sacrificial Cr disks and lifting off the PMGI layer. c) Normalize relative change in the stiffness of a surface patterned with a central PAcrAM-g-PEG-N3, surrounded by PAcrAM-g-PEG-Biotin ring and backfilled with PAcrAM-g-PMOXA in between the nanostructures. Data are obtained by a Peak Force QNM measurements in liquid.

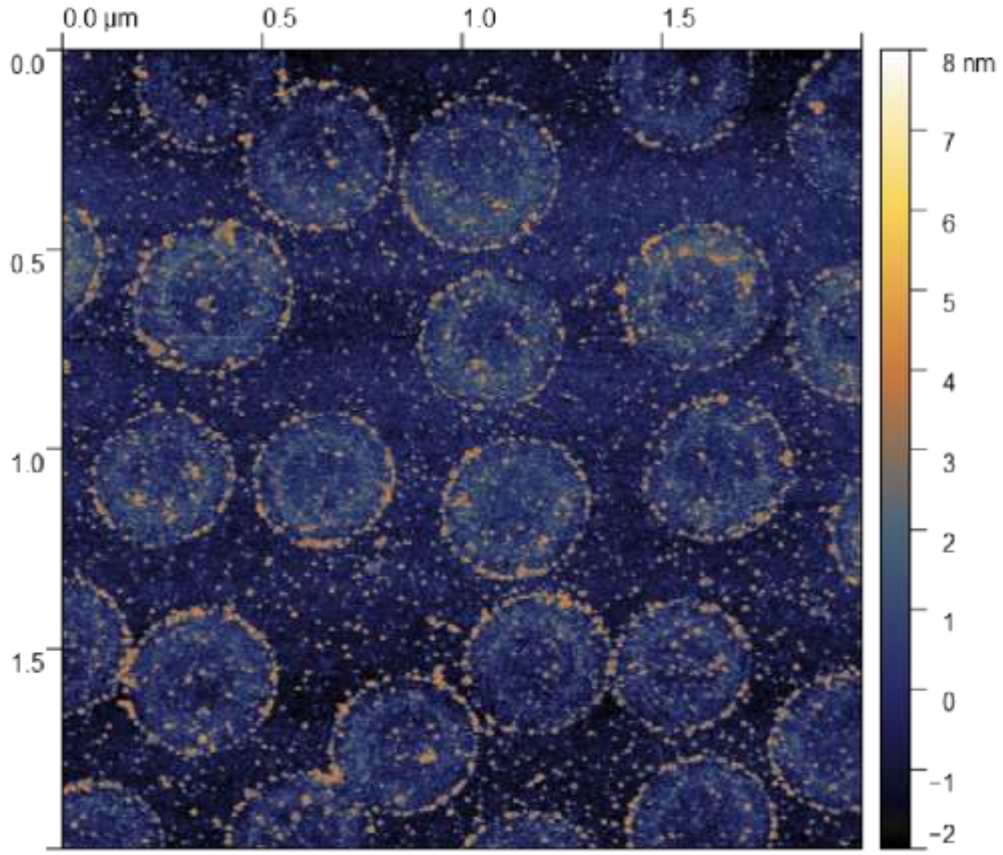

Figure S8 Liquid AFM scan of the 200 nm patterned surface prior to incubation with proteins.

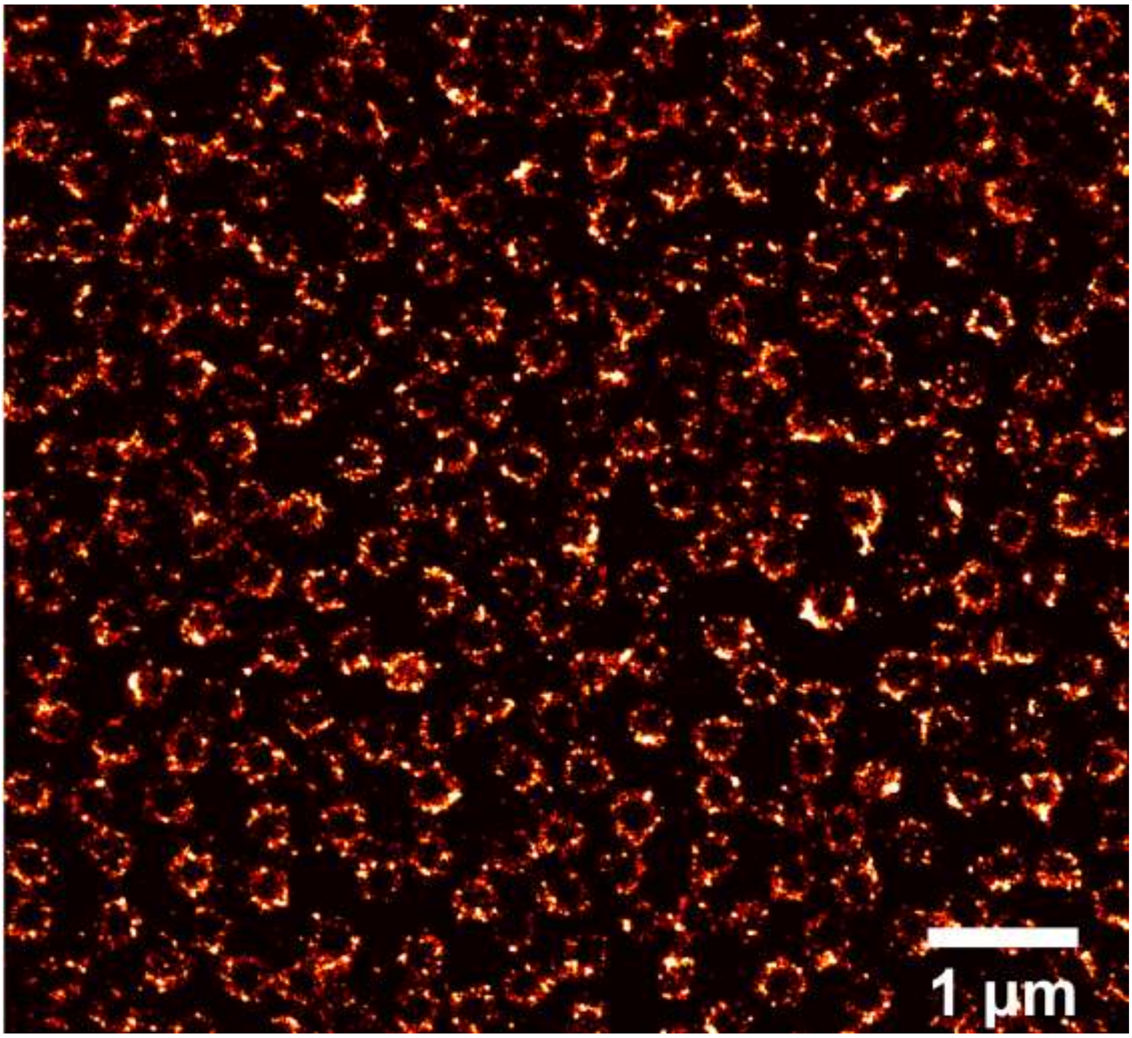

Figure S9 DNA-PAINT super-resolution image, showing the formation of 200 nm streptavidin rings

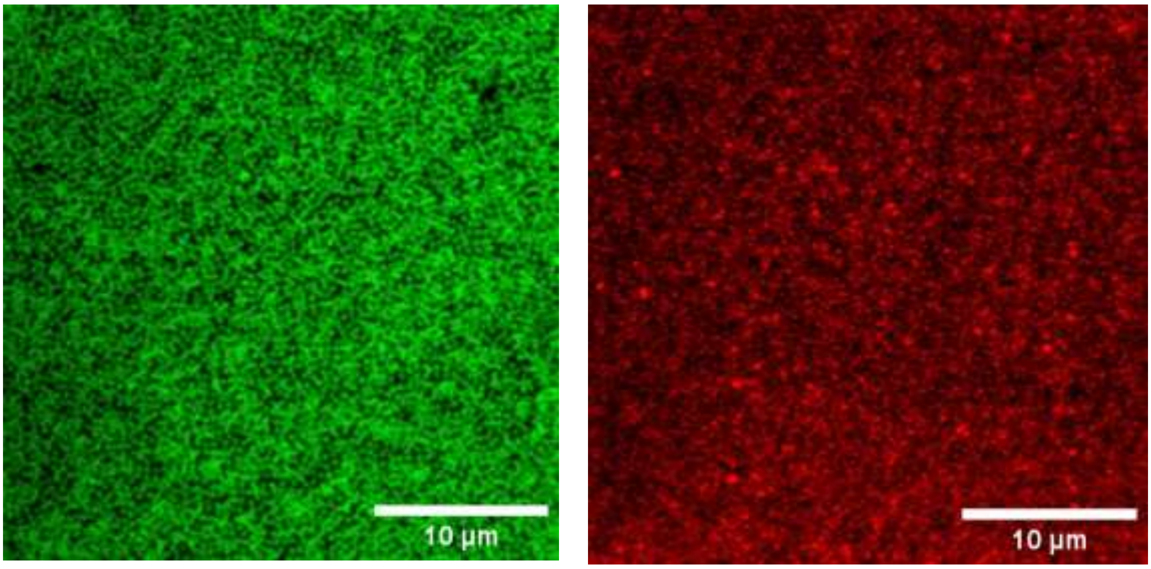

Figure S10 Fluorescence image of dual ligand protein pattern formation on 210 nm nanostructures. Green streptavidin, red DBCO-BSA. (zoom-in)

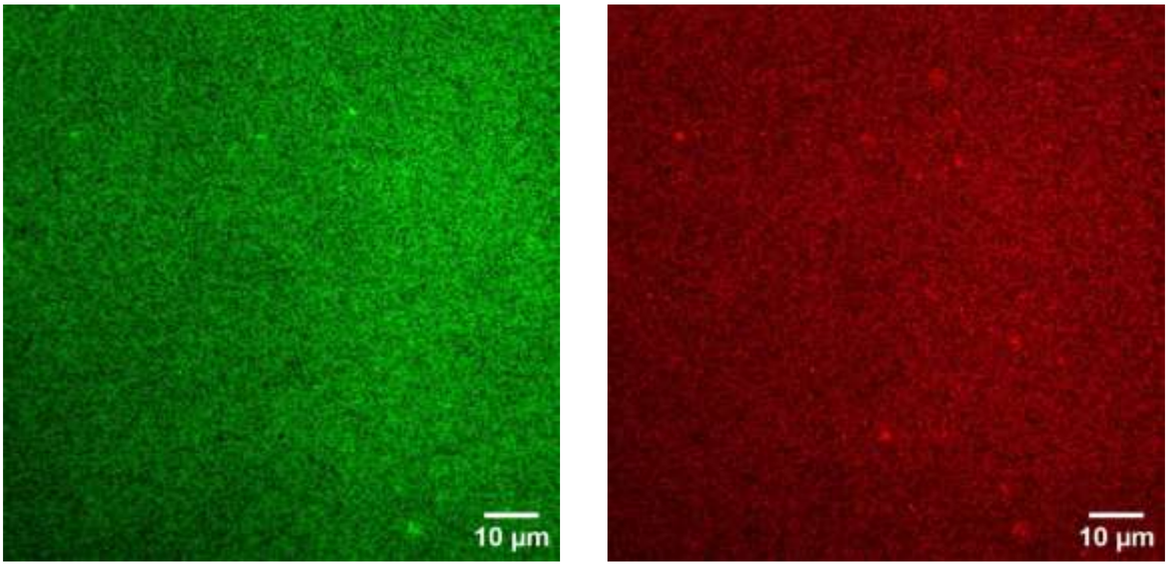

Figure S11 Fluorescence image of dual ligand protein pattern formation on 210 nm nanostructures. Green Streptavidin, red DBCO-BSA. (Overview)

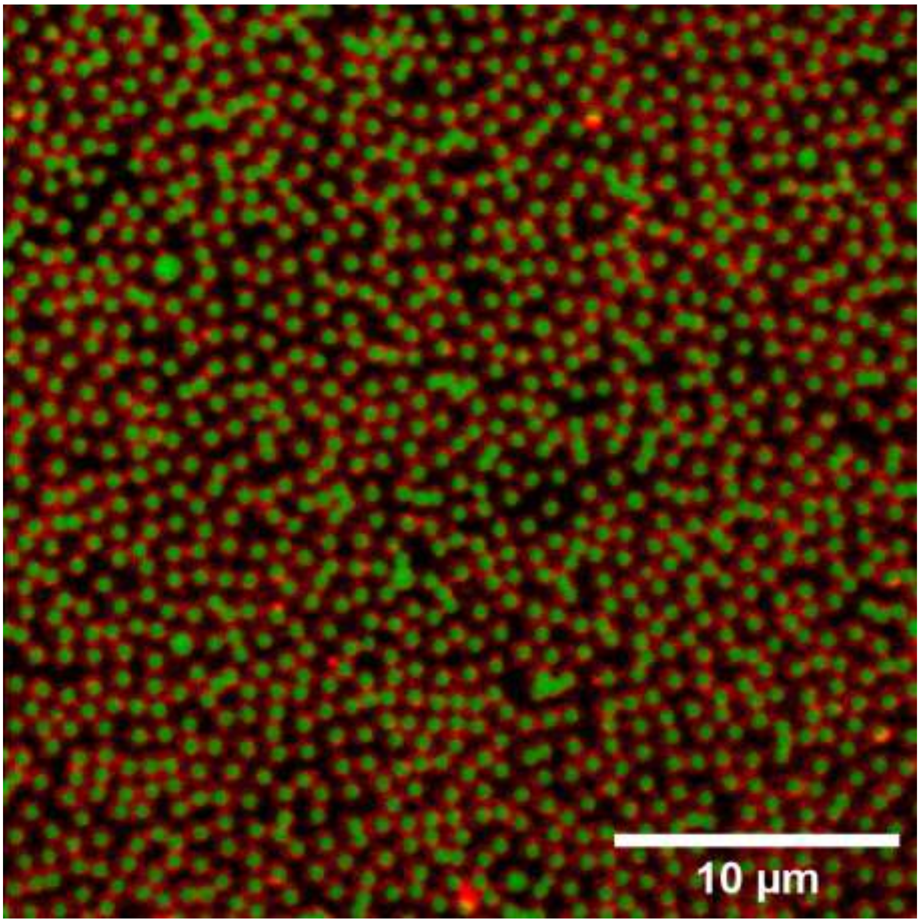

Figure S12 Swapping the deposition order of the biospecific PEG-Biotin and PEG-N_3 polymers. Red DBCO-BSA, green streptavidin.

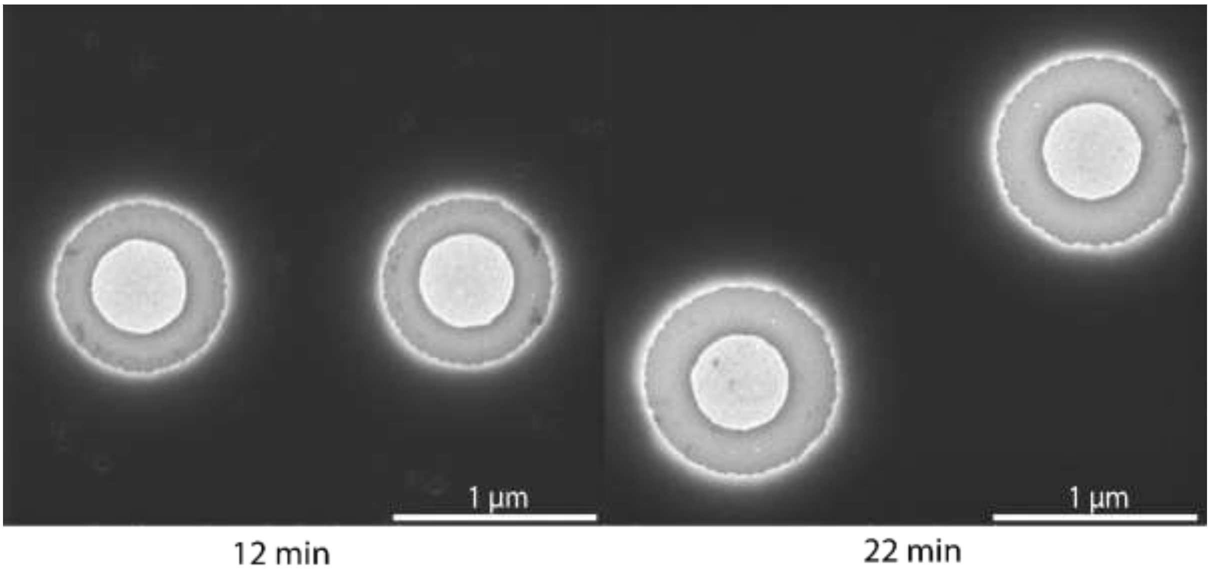

Figure S13 SEM images showing the effect of different O_2 RIE etching times on the ring-region dimension for 470 nm nanoparticles. The PMGI layer was covered with a thin 20 nm PMMA layer, which was lifted off after etching to reveal the ring region.

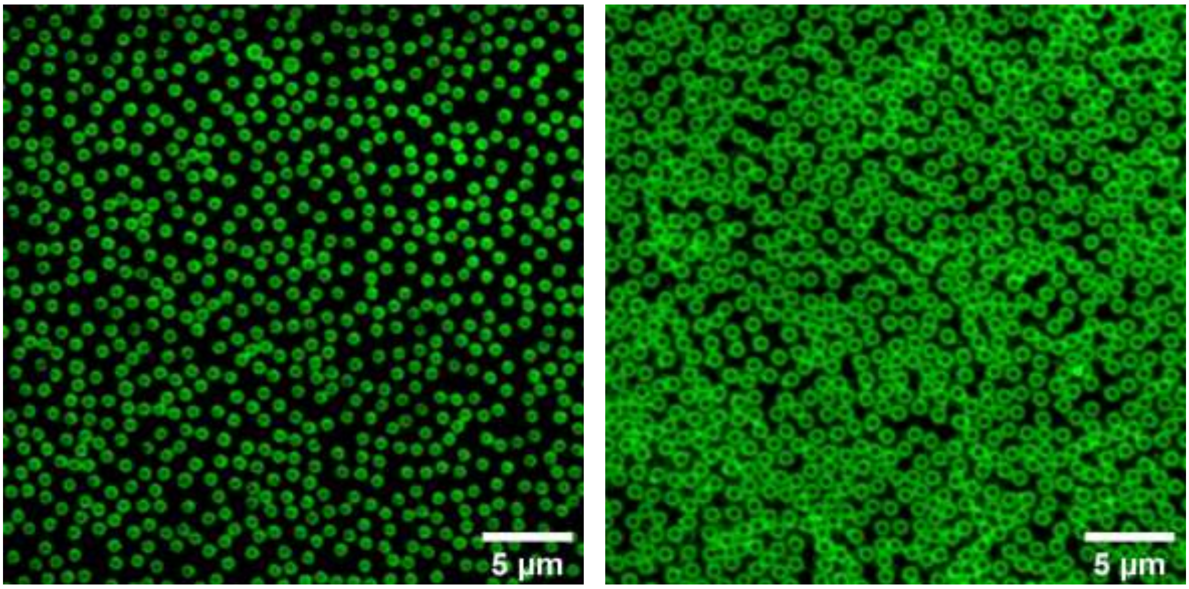

10 min etch **15 min etch**

Figure S14 Streptavidin nano-ring formation on 500 nm structures, etched for different durations as described in Figure S12.

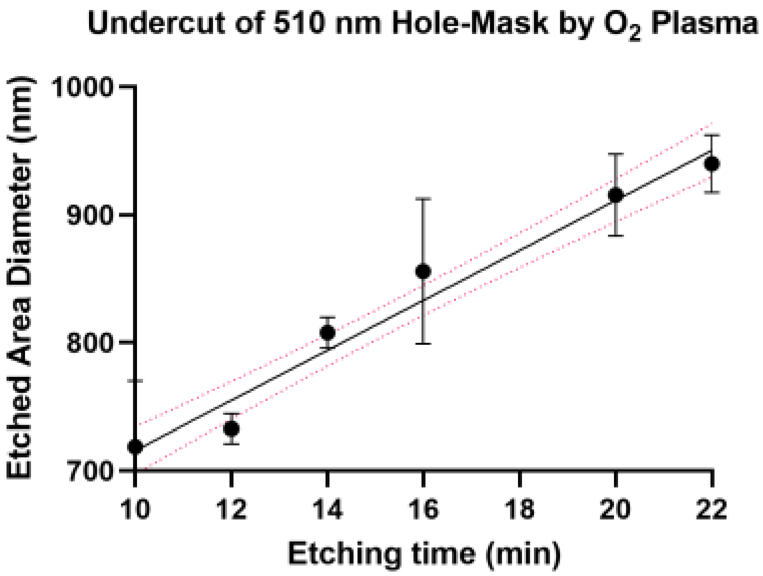

Figure S15 Data points and linear fit to determine the trend for increase in etching time *vs.* diameter of the ring region.

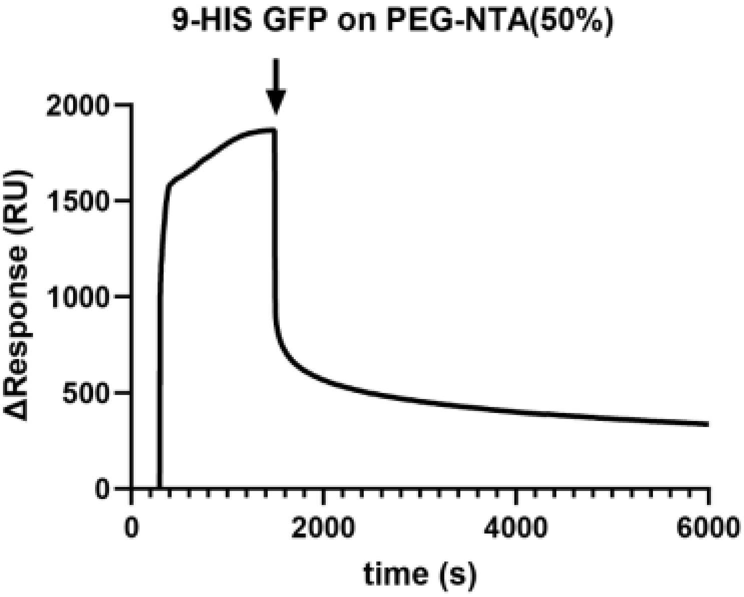

Figure S16 SPR sensorgram, showing the rapid desorption of 9-his GFP from PLL-g-PEG-NTA(50%) surface, pre-activated with Ni^{2+}.

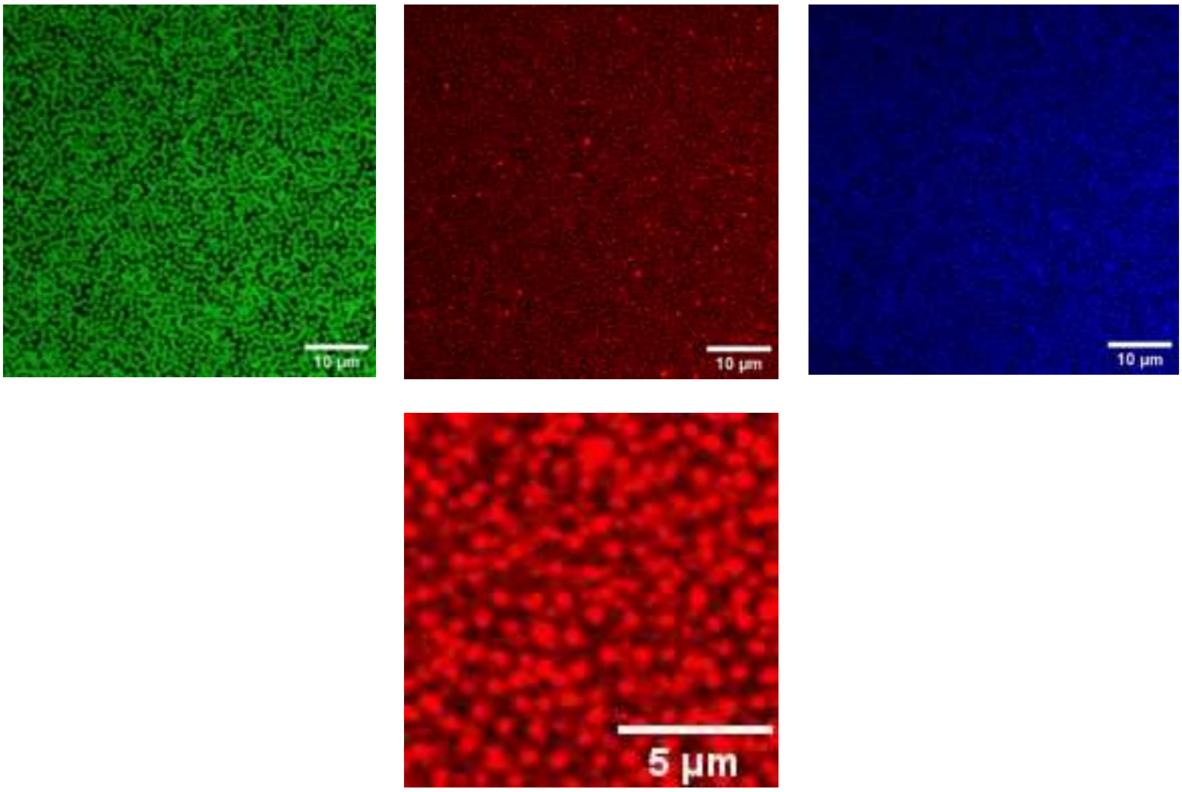

Figure S17 Example showing the nonspecific binding of proteins to the background region in tri-ligand patterning without anti-fouling polymer in the background. *i.e.*, step h and i emitted in Figure S6. Green AF488-Streptavadin, Red DBCO-BSA-Cy3, blue, BSA-Cy5. The nonspecific binding between the nanopatterns is clear when zoomed in on the red channel (DBCO-BSA-Cy3), and the contrast is enhanced.

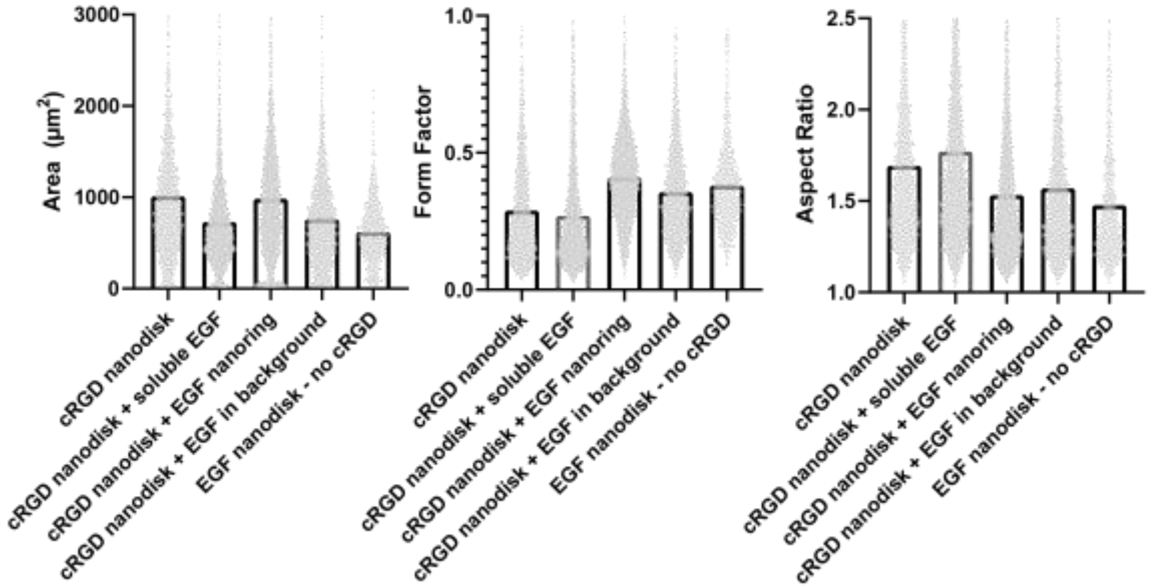

Figure S187 The plots represent the spread area, form factor (4·π·Area/Perimeter^2), and aspect ratio (maximum Feret diameter / minimum Feret diameter) for 2000-4000 cells in each condition; the bar represents the mean of all the individual cells. Negligible cell binding was detected on the PEGylated surfaces lacking EGF and cRGD (data not shown).

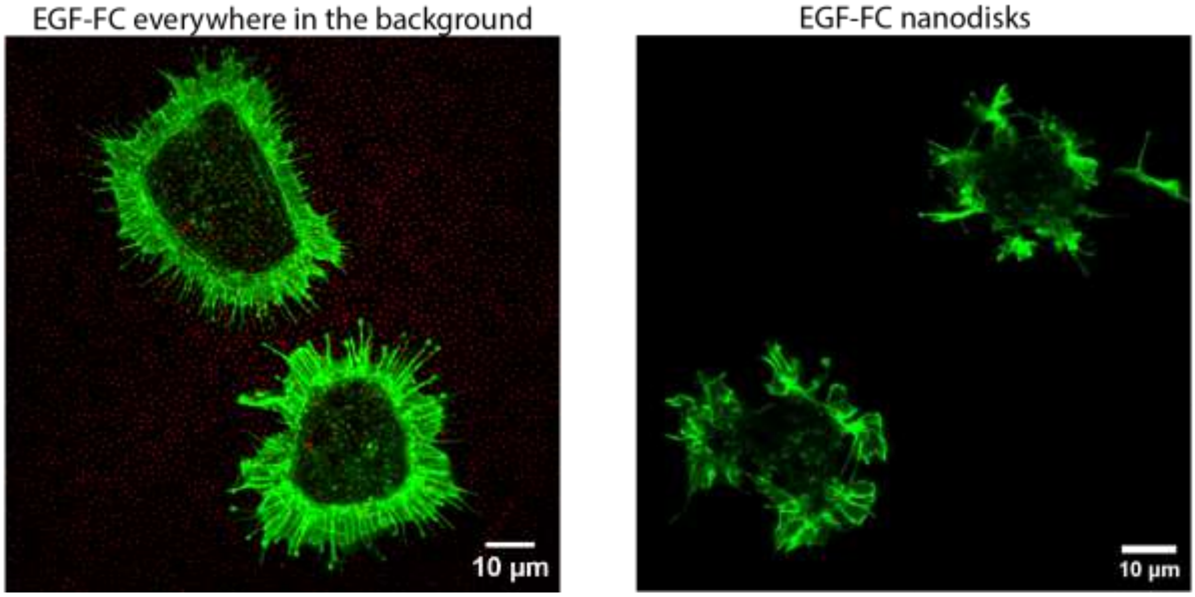

Figure S19 Left: Representative F-actin (green) and tension (red) fluorescent image of 3T3 fibroblasts on 800 nm cRGD TGTs. The fluorescent image represents cells interacting with substrates with cRGD-TGT DNA conjugated nanodisks and EGF everywhere in between them (not only ring-region). Right: cells interacting with nanopatterned EGF without cRGD TGTs.

Size	Catalog No. (Thermo Fischer)	LOT No.	Nominal Size	Coefficient of Variation of Diameter	Surface Charge Density
**200 nm**	S37491	2203280	210 nm	4.9%	0.5 $\mu C/cm^2$
**500 nm**	S37494	1537639	510 nm	6.3%	0.7 $\mu C/cm^2$
**600 nm**	S37495	1724527	600 nm	2.2%	9.1 $\mu C/cm^2$
**800 nm**	S37497	1650431	810 nm	2.0%	5.2 $\mu C/cm^2$

Table S1 Specifications of the colloidal nanoparticles used. Negatively charged sulfate latex beads are used to pattern the substrates. The particles' LOT number, nominal sizes, coefficient of variation of diameter, and surface charge density are reported from the manufacturer's product information.
